# Three Iodoargentate-Based Hybrids Decorated by Metal Complexes: Structures, Optical/Photoelectric Properties and Theoretical Studies

**DOI:** 10.3390/molecules28166116

**Published:** 2023-08-18

**Authors:** Minghui Liu, Xiaochen Ren, Weiyang Wen, Baohan Li, Jiaqi Li, Jun Li, Bo Zhang

**Affiliations:** 1 Department of Chemistry and Chemical Engineering, Liaocheng University, Liaocheng 252059, China; mhliu_lcu@163.com (M.L.); renxiaochen777@163.com (X.R.); bhli_lcu@163.com (B.L.); jiaqili_lcu@163.com (J.L.); 2 State Key Laboratory of Structural Chemistry, Fujian Institute of Research on the Structure of Matter, Chinese Academy of Sciences, Fuzhou 350002, China; wenweiyang@fjirsm.ac.cn

**Keywords:** iodoargentate, metal complex cation, structure, optical behavior, photocurrent response, theoretical study

## Abstract

So far, the development of new iodoargentate-based hybrids, especially those compounds with metal complex cations, and the understanding of their structure–activity relationships have been of vital importance but full of challenges. Herein, using the in-situ-generated metal complex cations as structural directing agents, three new iodoargentate-based hybrids, namely, [Co(phen)_3_]Ag_2_PbI_6_ (phen = 1,10-phenanthroline; **1**), [Ni(5,5-dmpy)_3_]Ag_7_I_9_·CH_3_CN (5,5-dmpy = 5,5-dimethyl-2,2-bipyridine; **2**) and [Co(5,5-dmpy)_3_]Ag_5_I_8_ (**3**), have been solvothermally prepared and then structurally characterized. Compound **1** represents one new heterometallic Ag–Pb–I compound characteristic of the chain-like [Ag_2_PbI_6_]*_n_*^2*n*−^ anions. Compound **2** features the straight one-dimensional (1D) [Ag_7_I_9_]*_n_*^2*n*−^ anionic moieties, while compound **3** contains infrequent two types of curved [Ag_5_I_8_]*_n_*^3*n*−^ anions. Optical properties reveal that the title compounds exhibit interesting semiconductor behaviors with the band gaps of 1.59–2.78 eV, which endow them with good photoelectric switching performances under the alternate light irradiations. We also present their Hirshfeld surface analyses, and the theoretical studies (band structures, density of states (DOS) and partial density of states (PDOS)).

## 1. Introduction

Organic–inorganic hybrid metal halides continue to fascinate researchers, which not only profit from their diversified structures, but also from their unusual photophysical performances inherited from the synergistic combinations of both organic and inorganic components [1,2,3]. Among them, iodoargentate-based hybrids are of special interest by virtue of the wide applications in many fields, such as white light emission, thermo-/photo-chromism, photocurrent response, dye sorption/separation and photocatalysis, gaining more and more attention in recent years [4,5,6,7,8,9].

From the standpoint of anionic moieties, Ag^+^ ion possesses the flexible coordination modes comprising the [AgI_2_] dumbbell, [AgI_3_] triangle, [AgI_4_] tetrahedron and [AgI_6_] octahedron [10,11]. More attractively, these primary building units also feature high self-assembly characterizations, which provide the abundant possibilities of generating numerous secondary building units (SBUs). By sharing of corner/edge/face, together with the short interactions of Ag⋯Ag, a large number of SBUs, such as [Ag_5_I_6_]^−^, [Ag_3_I_7_]^4−^, [Ag_5_I_9_]^4−^, [Ag_6_I_10_]^4−^, [Ag_6_I_11_]^5−^, [Ag_3_I_8_]^5−^, [Ag_5_I_10_]^5−^, [Ag_7_I_12_]^5−^, [Ag_4_I_10_]^6−^, [Ag_6_I_12_]^6−^, [Ag_8_I_14_]^6−^, [Ag_11_I_18_]^7−^, [Ag_10_I_17_]^7−^, and [Ag_10_I_18_]^8−^ have been successively found [10,11]. From the perspective of cationic components, hitherto, multiple types of templates (e.g., aliphatic amines, aromatic amines and metal complexes) have been broadly exploited to decorate and prepare the novel iodoargentate-based hybrids, which range from the zero-dimensional (0D) discrete clusters, one-dimensional (1D) chains, two-dimensional (2D) layers, and three-dimensional (3D) frameworks [10,11]. Of these, the metal complex cations have received extensive research interest due to the stable and strong structural directing effects. More importantly, most of them are optically active or photosensitive, and their introduction may endow the as-synthesized materials with intriguing physical and chemical properties. Representative examples are [M(bipy)_3_]Ag_3_I_5_ (M = Zn, Ni, Co, Fe, Mn; bipy = 2,2-bipyridine), K[M(bipy)_3_]_2_Ag_6_I_11_ (M = Zn, Ni, Co, Fe, Mn), [(Ni(bipy)_3_][H-bipy]Ag_3_I_6_, [M(bipy)_3_]Ag_5_I_7_ (M = Co, Zn, Ni), [Co(bipy)_3_]Ag_3_I_6_, {[Ni(bipy)(THF)_2_(H_2_O)_2_](Ag_10_I_12_)_3_·2DMF}*_n_* (THF = tetrahydrofuran, DMF = *N*,*N*-dimethylformamide), [Co(phen)_3_]Ag_2_I_4_·3DMF, and [Cd(phen)_3_]_2_Ag_13_I_17_ [9,12,13,14,15,16,17]. In addition, some heterometallic phases including [Co(bipy)_3_]AgPb_2_I_7_, [Ni(phen)_3_]Ag_2_PbI_6_, [Ni(bipy)_3_]AgBiI_6_, [Co(bipy)_3_]_2_Ag_4_Bi_2_I_16_, etc., have also been isolated [18,19,20,21]. Among them, compound K[Fe(bipy)_3_]_2_Ag_6_I_11_ exhibits the visible light-driven photocatalytic decontamination of organic pollutant, while compound [Ni(bipy)_3_]AgBiI_6_ displays the photocurrent response behavior under the alternating light radiation [13,20]. It is worth noting that most existing metal complex-decorated iodoargentate-based hybrids focus on the bipy ligands. Relatively speaking, the instances with other types of ligands are rarely documented. Therefore, enriching the new members of metal complex-templated iodoargentate family and further exploring their structure–activity relationships are still of great significance but challenging.

In general, the former transition metals (e.g., Ni, Co, Mn, and Fe) are easy to be coordinated by the chelated amines or organic ligands. As such, with the in-situ-formed metal complexes as charge-compensating agents, we successfully isolated three new iodoargentate-based hybrids, namely [Co(phen)_3_]Ag_2_PbI_6_ (**1**), [Ni(5,5-dmpy)_3_]Ag_7_I_9_·CH_3_CN (**2**) and [Co(5,5-dmpy)_3_]Ag_5_I_8_ (**3**). Further studies show that the title compounds possess the chain-like structures, exhibiting the semiconductor behaviors with the optical band gaps of 1.59–2.78 eV. In addition, they also display rapid and stable photoelectric converting performances under the alternate light illuminations, with photocurrent densities comparable to those of many halide analogues. This work also provides Hirshfeld surface analyses and theoretical results.

## 2. Results and Discussion

### 2.1. Crystal Structures

#### 2.1.1. Structural Description of [Co(phen)_3_]Ag_2_PbI_6_ (**1**)

**Figure 1:**
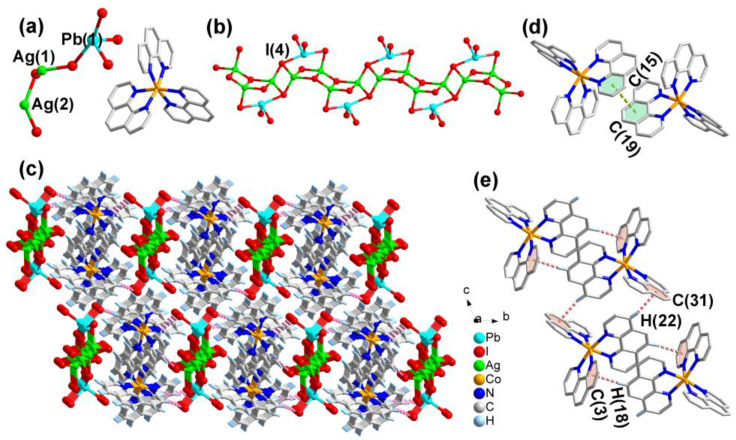
(**a**) The asymmetric unit of compound **1**. (**b**) The [Ag_2_PbI_6_]*_n_*^2*n−*^ anionic chain. (**c**) The packing diagram viewed along *a* axis; dashed lines show the interactions of C–H⋯I. The [Co(phen)_3_]^2+^ complexes showing the *π*⋯*π* (**d**) and C–H⋯*π* (**e**) interactions.

X-ray crystallographic analyses showed that compound **1** has the 1D [Ag_2_PbI_6_]*_n_*^2*n*−^ anionic chains that were charge balanced by the [Co(phen)_3_]^2+^ cations (Figure 1). The asymmetric unit of compound **1** is described in Figure 1a, consisting of one in-situ-generated [Co(phen)_3_]^2+^ cation, two Ag^+^ ions, one Pb^2+^ ion and six I^−^ ions. In **1**, Co^2+^ ions feature the octahedral coordination configurations, with Co–N bond lengths and N–Co–N angles of 2.134(13) to 2.198(13) Å and 76.9(5) to 168.3(6)°, respectively. In the 1D anionic moiety of [Ag_2_PbI_6_]*_n_*^2*n*−^, each Ag atom is 4-coordinated, giving the distorted tetrahedral configuration. The Ag–I bond lengths and I–Ag–I angles vary from 2.780(2)–2.991(2) Å and 98.14(7)–124.21(8)°, respectively. The Pb^2+^ ions lie in the *ψ*-{PbI_4_} coordinated environment with Pb–I bond lengths of 2.832(19)–3.4246(15) Å and I–Pb–I bond angles of 89.32(4)–162.62(4)°, respectively. Interestingly, the I atoms exhibit three coordination modes: I(2) and I(3) act as the terminal atoms, I(4) is the *μ*_3_-I atom connecting two Ag^+^ ions and one Pb^2+^ ion, and I(1), I(5) and I(6) serve as bridging modes linking two metal centers (Figure 1b). The above-mentioned coordination styles and observed values are normal and are comparable to some related analogues, such as [(Me)_2_DABCO]Ag_2_PbBr_6_, [Ni(bipy)_3_]AgPb_2_Br_7_ and [La(DMSO)_8_]Ag_2_Pb_2_I_9_ [18,22,23].

As presented in Figure 1b, two [AgI_4_] tetrahedra are condensed by edge-sharing to form the [Ag_2_I_6_] dimer. Noteworthily, the distance of Ag⋯Ag in the [Ag_2_I_6_] dimer reaches 3.234(4) Å, which is less than the sum of the van der Waals radii of Ag (3.44 Å), suggesting the significant metal–metal interactions [24]. This phenomenon is also observed in the case of [Fe(bipy)_3_]AgBiI_6_, [Zn(phen)_3_]_2_Ag_2_Bi_2_I_12_, [Co(bipy)_3_]_2_Ag_4_Bi_2_I_16_, [Ce(DMF)_8_]_2_Ag_10_Pb_3_I_22_, [Ni(bipy)_3_]AgPb_2_I_7_, [Fe(H_2_O)_6_]Ag_15_I_18_, [Cd(phen)_3_]_2_Ag_13_I_17_ and [Co(bipy)_3_]Ag_3_I_6_ [15,17,18,20,21,25,26,27]. Then, one [Ag_2_I_6_] unit joins two *ψ*-{PbI_4_} blocks through the I(4) atoms to generate the [Ag_2_Pb_2_I_12_]^6−^ moiety. Every two adjacent [Ag_2_Pb_2_I_12_]^6−^ units are further connected by the [Ag_2_I_4_] units to result in the curve-like [Ag_2_PbI_6_]*_n_*^2*n*−^ anionic chain extended along the *a*-axis. The in-situ-produced [Co(phen)_3_]^2+^ complexes serve as the structural directors and charge balancers, separating the anionic components and forming the 3D supramolecular framework through the extensive hydrogen bond contacts (Figure 1c). The C–H⋯I hydrogen bond lengths and C–H⋯I hydrogen bond angles are between 3.78(2)–3.968(16) Å and 129.9–155.0°. In addition to the C–H⋯I hydrogen bonds, compound **1** also contains the *π*⋯*π* and C–H⋯*π* interactions (Figure 1d,e).

#### 2.1.2. Structural Description of [Ni(5,5-dmpy)_3_]Ag_7_I_9_·CH_3_CN (**2**)

**Figure 2:**
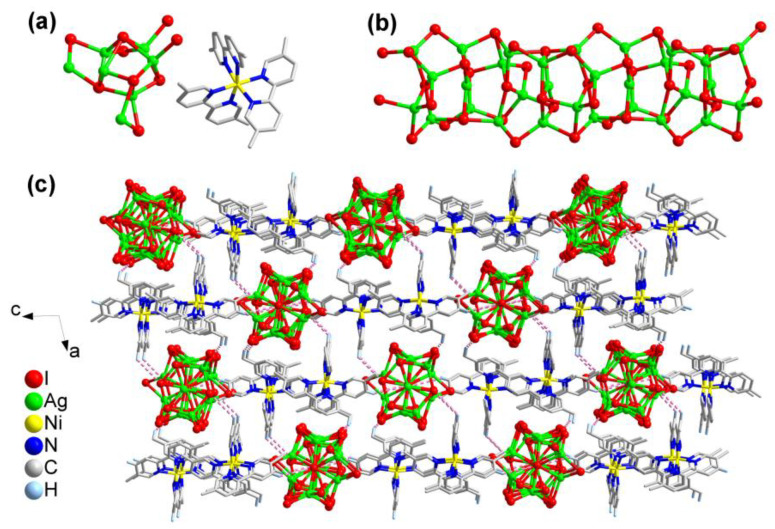
(**a**) One fragment for showing the asymmetric unit of **2**. (**b**) The [Ag_7_I_9_]*_n_*^2*n−*^ chain in **2**. (**c**) The packing diagram of **2** along the *b* axis, in which the guest CH_3_CN molecules are omitted for clarity and dashed lines are used for demonstrating the C–H⋯I interactions.

X-ray crystallographic studies revealed that compound **2** features the straight 1D [Ag_7_I_9_]*_n_*^2*n*−^ anionic chains that were isolated by the [Ni(5,5-dmpy)_3_]^2+^ metal complexes (Figure 2). The asymmetric unit of compound **2** includes one formula unit, that is, seven Ag^+^ ions, nine I^−^ ions, one in-situ-formed [Ni(5,5-dmpy)_3_]^2+^ cation and one free CH_3_CN molecule (Figure 2a). In the anionic moiety of [Ag_7_I_9_]*_n_*^2*n*−^, except for the triangular Ag(4) atom, the other Ag^+^ ions adopt the distorted tetrahedral coordination styles, with the Ag–I bond distances and I–Ag–I angles lying in the range of 2.6972(19)–3.1850(2) Å and 87.86(5)–130.84(7)°, respectively. For I atoms, interestingly, three different coordination modes are presented in compound **2**: I(1), I(7) and I(9) act as the bridging atoms; I(4), I(6) and I(8) are the *μ*_3_-I atoms linking three Ag^+^ ions; I(2), I(3) and I(5) are the *μ*_4_-I atoms that were bonded to four Ag^+^ ions. Like the case of **1**, the cationic metal centers are surrounded by six N atoms from three organic ligands, with Ni–N bond lengths and N–Ni–N angles of 2.0310(9) to 2.1010(11) Å and 78.6(4) to 177.2(4)°, respectively.

As shown in Figure S2a, edge-sharing of four [AgI_4_] tetrahedra to produce the [Ag_4_I_9_] unit, which was encapsulated by two [AgI_4_] tetrahedra and one [AgI_3_] triangle by sharing the I(2), I(3), I(4) and I(5) atoms to obtain the [Ag_7_I_13_] moiety (Figure S2b). In the [Ag_7_I_13_] moiety, noteworthily, the shortest Ag⋯Ag separation achieves 2.9610(2) Å, implying the obvious d^10^-d^10^ argentophilic interactions [24]. Every two centrosymmetrical [Ag_7_I_13_] moieties are further condensed by the I(1), I(2), I(7) and I(8) atoms to give the [Ag_14_I_22_] moieties (Figure S2c), which further link the adjacent ones to get the complex [Ag_7_I_9_]*_n_*^2*n−*^ anionic chains that straightly extended along the *b* axis (Figure 2b). The [Ag_7_I_9_]*_n_*^2*n*−^ anionic moieties collaborate with the [Ni(5,5-dmpy)_3_]^2+^ cations and lattice CH_3_CN molecules, resulting in the 3D supramolecular framework with the help of extensive C–H⋯I hydrogen bonds (Figure 2c). The C–H⋯I hydrogen bond lengths and C–H⋯I hydrogen bond angles scatter from 3.857(13)–3.912(13) Å and 123.2–147.1°, respectively.

#### 2.1.3. Structural Description of [Co(5,5-dmpy)_3_]Ag_5_I_8_ (**3**)

**Figure 3:**
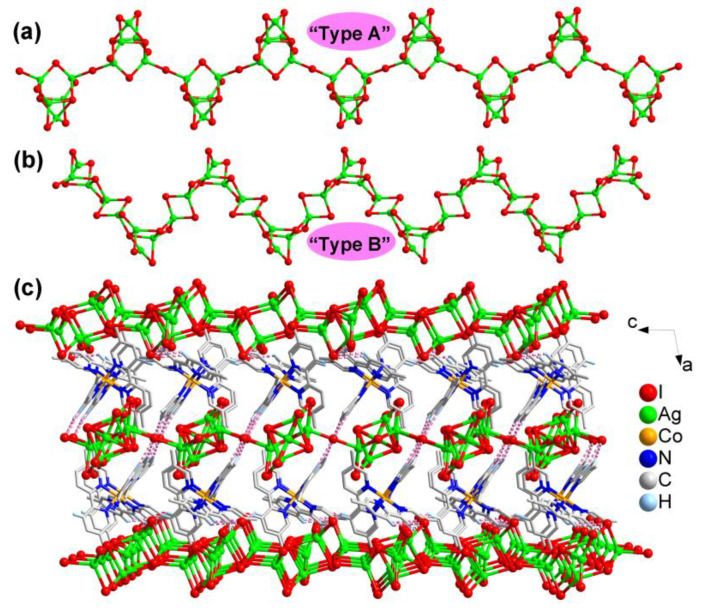
(**a**) The [Ag_5_I_8_]*_n_*^3*n−*^ anionic chain, denoted as “type A”. (**b**) The [Ag_5_I_8_]*_n_*^3*n−*^ anionic chain, denoted as “type B”. (**c**) Perspective view of compound **3** along the *b* axis; dashed lines show the interactions of C–H⋯I.

Compound **3** exhibits two types of curved [Ag_5_I_8_]*_n_*^3*n*−^ anions (Figure 3). As described in Figure S3, its asymmetric unit was composed of four Ag^+^ ions (Ag(2), Ag(3), Ag(4) and Ag(5)), two halves of Ag^+^ ions (Ag(1) and Ag(6)), seven I^−^ ions (I(1), I(2), I(3), I(6), I(7), I(8) and I(9)), two halves of I^−^ ions (I(4) and I(5)) and one in-situ-formed [Co(5,5-dmpy)_3_]^3+^ cation. In compound **3**, except for the Ag(6), the other Ag^+^ ions are surrounded by four I^−^ ions, while the I^−^ ions present two different bonding patterns (I(1), I(3), I(4), I(5), I(6), I(8) and I(9) are *μ*_2_-I atoms; I(2) and I(7) are *μ*_4_-I atoms). The coordination modes of Co^3+^ ions, the observed bond lengths of Ag–I and Co–N, and the angles of I–Ag–I and N–Co–N are also unexceptional and coincide with those in the literature [9,13,15].

Compound **3** exhibits a captivating structural characteristic, wherein it encompasses two distinct types of anionic chains. As depicted in Figure S4a, face-sharing of three [AgI_4_] tetrahedra to produce a [Ag_3_I_6_] trimer, which connects a [Ag_2_I_7_] unit by I(2) and I(3) atoms to form a [Ag_5_I_9_] moiety. Every two neighboring [Ag_5_I_9_] moieties are interconnected via I(5) atoms to obtain the [Ag_5_I_8_]*_n_*^3*n−*^ anionic chain denoted as “type A” (Figure 3a). Two [AgI_4_] tetrahedra and one [AgI_3_] triangle are condensed by I(7) and I(9) atoms to generate another [Ag_3_I_6_] trimer, which connects two [AgI_4_] tetrahedra through the edge-sharing to create the [Ag_5_I_10_] unit (Figure S4b). Such [Ag_5_I_10_] units are further interconnected via edge-sharing to result in the final [Ag_5_I_8_]*_n_*^3*n−*^ anionic chain denoted as “type B” (Figure 3b). The [Co(5,5-dmpy)_3_]^3+^ cation acting as the charge-balancing agents isolated the above-mentioned two types of anionic chains, forming the extensive C–H⋯I hydrogen bonding interactions, with the bond lengths and angles in the range of 3.71(2)–4.00(2) Å and 127.6–172.5°, respectively (Figure 3c).

### 2.2. Hirshfeld Surface Analyses

**Figure 4:**
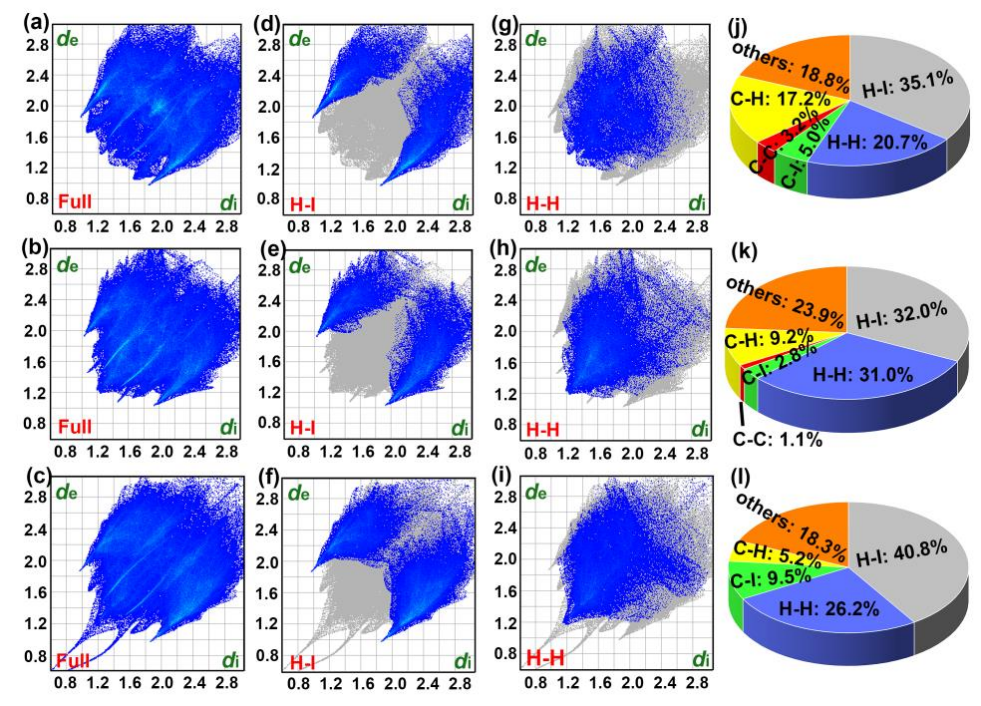
The 2D fingerprint plots for title compounds. Total interactions ((**a**), **1**; (**b**), **2**; (**c**), **3**). H⋯I interactions ((**d**), **1**; (**e**), **2**; (**f**), **3**). H⋯H interactions ((**g**), **1**; (**h**), **2**; (**i**), **3**). Contribution percentages of various interactions existing in title compounds ((**j**), **1**; (**k**), **2**; (**l**), **3**).

In order to visually and quantitatively determine the percentage of the area occupied by different types of intermolecular interactions, we performed the Hirshfeld surface analyses for title compounds. The full interactions were depicted in Figure 4a–c, with a scattering range of 2.6 ≤ *d_e_* + *d_i_* ≤ 5.8 Å for **1**, 2.4 ≤ *d_e_* + *d_i_* ≤ 5.8 Å for **2**, 2.0 ≤ *d_e_* + *d_i_* ≤ 5.8 Å for **3**, respectively. The decomposed 2D fingerprint plots showed that the H⋯I/I⋯H hydrogen bonds emerged like two wings of a bird, which accounted for the highest proportion of the total Hirshfeld surface area (35.1% for **1**, 32.0% for **2** and 40.8% for **3**; Figure 4d–f). For compound **1**, the ratio of H⋯I contacts is 13.9% (top left), which is smaller than the ratio of I⋯H contacts (21.2%, bottom right). Similar case has also been observed in compounds **2** and **3**. Further investigations revealed that the second contributors are due to the H⋯H contacts. As shown in Figure 4g–i, they mainly occurred in the intermediate region, making up 20.7% (**1**), 31.0% (**2**) and 26.2% (**3**) of the total surface. Furthermore, the crystal packing of compound **1** is significantly influenced by the C⋯H/H⋯C and C⋯C contacts. In general, these contacts are frequently employed to highlight the C–H⋯*π* (17.2%) and *π*⋯*π* (3.2%) interactions, thus underscoring their vital significance in the overall structure (Figure S5a,c). Although compound [Ni(phen)_3_]Ag_2_PbI_6_ is isomorphic with **1**, the C–H⋯*π* and *π*⋯*π* interactions have different contribution proportions, accounting for 10.0% and 16.0% of total Hirshfeld surface, respectively [19]. It is noteworthy that the *π*⋯*π* interactions are not obvious in compounds **2** and **3**. These findings are consistent with the results of crystal structure analyses. The comparative contributions from other interactions are illustrated in Figure 4j–l. Clearly, the interactions associated with hydrogen atoms contribute greatly to their structural stabilities. The occurrence of this phenomenon is unsurprising and frequently observed in numerous metal halides, like [NH_4_][Fe(bipy)_3_]_2_[Ag_6_Br_11_], [Zn(bipy)_3_]_2_Ag_2_BiI_6_(I)_1.355_(I_3_)_1.645_, [Fe(phen)_3_]_2_Ag_3_Pb_2_I_11_, [Ni(phen)_3_]Ag_2_PbI_6_ and [Ni(5,5′-dmbpy)_3_]_2_Ag_4.9_I_8.9_·4H_2_O [19,28,29,30,31]. More Hirshfeld surface comparisons of compounds **1**–**3** with some related analogues are listed in Table S10.

### 2.3. Optical Properties

**Figure 5:**
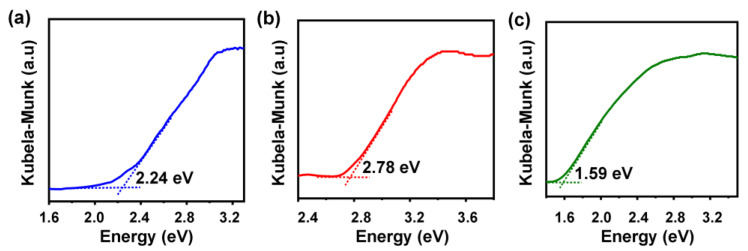
The optical diffuse reflectance patterns (**1** (**a**), **2** (**b**) and **3** (**c**)).

The UV–Vis diffuse reflectance spectra, obtained from powder samples at room temperature, show that the optical absorption edges of title compounds are estimated to be 2.24 eV for **1**, 2.78 eV for **2**, and 1.59 eV for **3**, respectively (Figure 5a–c). This indicates that the title compounds are potential visible light responsive semiconductors. In addition, these band values are very consistent with their respective crystal colors and match well with those of some reported Ag-based metal halide analogues, e.g., K[Fe(bipy)_3_]_2_Ag_6_I_11_ (1.66 eV), K[Co(bipy)_3_]_2_Ag_6_I_11_ (1.75 eV), [Fe(phen)_3_]_2_Ag_3_I_7_ (1.78 eV), [NH_4_][Fe(bipy)_3_]_2_Ag_6_Br_11_ (1.90 eV), [Ni(bipy)_3_]AgPb_2_I_7_ (1.92 eV), [Co(bipy)_3_]Ag_3_I_6_ (2.03 eV), [Ni(5,5-dmbpy)_3_]_2_Ag_4.9_I_8.9_·4H_2_O (2.07 eV), [(Me)_2_-DABCO]_2_Ag_5_Pb_2_I_13_ (2.33 eV), [Ni(dien)(MeCN)_3_]Ag_2_Pb_3_I_10_·MeCN (2.42 eV), [V(DMSO)_5_(H_2_O)]Ag_6_I_8_ (2.61 eV), [Ni(phen)_3_]Ag_2_I_4_·3DMF (2.64 eV) and [Co(phen)_3_]_2_Ag_11_I_15_·H_2_O (2.84 eV) [9,13,15,17,18,22,28,31,32,33,34].

### 2.4. Photocurrent Responses

**Figure 6:**
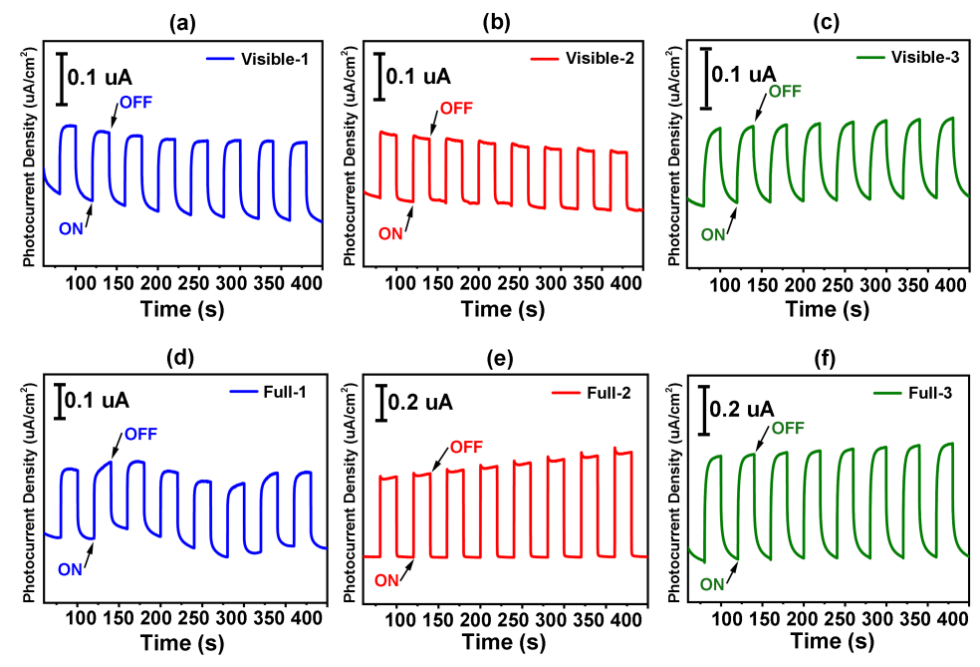
Photocurrent–time curves of visible light illumination for compounds **1** (**a**), **2** (**b**), and **3** (**c**), respectively. Photocurrent–time curves of full-spectrum light illumination for compounds **1** (**d**), **2** (**e**), and **3** (**f**), respectively.

Inspired by the semiconductor nature of title compounds, we have further tested their photoelectric performances in a KCl solution using a classic three-electrode configuration, which were commonly used to evaluate the potential applications in the photovoltaic field. The photocurrent–time curves with switching interval of 20 s were recorded in Figure 6. It can be seen that they have the obvious photocurrent response behaviors under the alternating light irradiation. The average visible light photocurrent densities of compounds **1**–**3** are 0.16, 0.14 and 0.14 μA cm^−2^ (Figure 6a–c), respectively, which are well comparable with some high-performance metal halides, such as [Ag_2_I_2_(phen)]*_n_*, {[Nd_2_(dpdo)(DMF)_14_](Ag_12_I_18_)}*_n_*, [Ni(phen)_3_]Pb_2_I_6_·CH_3_CN, [Co(bipy)_3_]_2_Pb_8_I_21_, {[La(dpdo)(DMF)_6_](Bi_2_I_9_)_2_}*_n_*, [Ni(bipy)_3_]AgBiI_6_, and [Zn(bipy)_3_]_2_Ag_2_BiI_6_(I)_1.355_(I_3_)_1.645_ [20,29,35,36,37,38]. High photocurrent densities indicate that they possess the satisfactory transfer capacity of charge carriers. In addition, there are no substantial declines of the photocurrent switching ratios after multiple cycles, which prove the excellent stability and repeatability. This is significantly superior to [CH_3_NH_3_]PbI_3_, a classic perovskite photovoltaic material, which usually became unstable due to the hydrolysis instability when exposed to the light irradiation. More importantly, their photoelectric switching abilities could be further enhanced with the conversion of visible light to the full spectrum condition (Figure 6d–f). Such a phenomenon is normal and has also been observed in the case of [NH_4_][Fe(bipy)_3_]_2_Ag_6_Br_11_, [Pb(MCP)_2_I]PbI_3_, [Zn(phen)_3_]_2_Ag_2_Bi_2_I_12_, and [Co(bipy)_3_]_2_Ag_4_Bi_2_I_16_ [21,25,28,39].

### 2.5. Theoretical Studies

**Figure 7:**
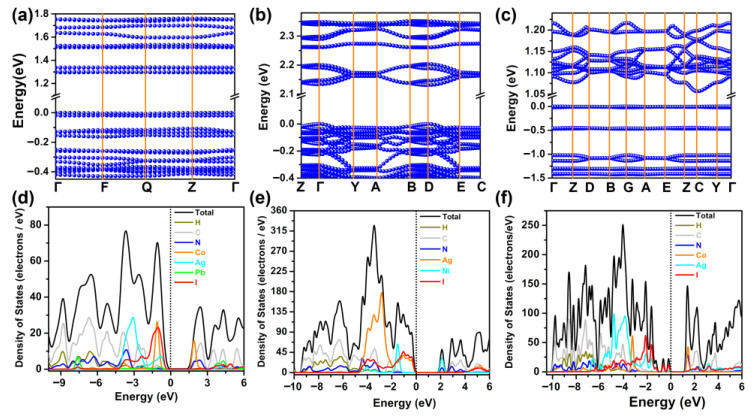
The band gaps of compounds **1** (**a**), **2** (**b**) and **3** (**c**). The DOS curves of compounds **1** (**d**), **2** (**e**) and **3** (**f**).

In order to gain a deeper understanding of the optical properties and photoelectric behaviors of title compounds, this study utilizes density functional theory (DFT) and the first-principles approach to analyze their electronic structures (Figure 7). According to the Figure 7a, the valence band (VB) maximum and the conduction band (CB) minimum of compound **1** are located at the same symmetric point (Γ), indicating the direct band gap semiconductor character. Compounds **2** and **3**, in contrast to **1**, are indirect band gap materials. In detail, the VB maximum are both situated at Γ point, while the CB minimum appear at the D for **2** and C points for **3**, respectively (Figure 7b,c). Specially, compounds **2** and **3** exhibit obvious band dispersions, generally meaning the small effective mass and the good carrier transport. According to theoretical calculations, the band gap values of compounds **1**–**3** are found to be 1.29, 1.82, and 1.06 eV, respectively. However, upon comparison with the experimental results of 2.24, 2.78, and 1.59 eV, it is evident that the theoretical values underestimate the true values. This underestimation is a common phenomenon and can be attributed to the limitations of DFT calculations [40,41].

By examining the DOS and PDOS diagrams of compound **1**, it can be observed that the bottom of CB is primarily influenced by the Co–3d, N–2p and C–2p orbitals through local interactions (Figure 7d and Figure S15). The VB near the Fermi level is mainly contributed by the 3d states of Co and Ag mixed with the 5p state of I. Note that the contribution of Pb^2+^ ions to the front orbitals near the Fermi level is negligible. In addition, the contribution in the region of −4 to −15 eV is almost from the C–2p and I–5s states. For compound **2**, the top part of VB mainly originates from 5p state of I and 3d states of Co and Ag, while the CB minimum is mostly constituted by Ni–3d with small amounts of N–2p states (Figure 7e and Figure S16). For compound **3**, the mixing of I–5p state makes up the bottom of the CB, and the VB is dominated by Co–3d, C–2p and N–2p states (Figure 7f and Figure S17). The results of this study indicate that the optical properties of the compounds mentioned are influenced by both organic and inorganic components, particularly the photosensitive metal complex cations. This discovery is consistent with previous studies on metal halides with optical activities, such as [Ni(phen)_3_]Ag_2_PbI_6_, [Fe(bipy)_3_]AgPb_2_Br_7_, [NH_4_][Ni(phen)_3_]BiI_6_, [Co(phen)_3_]Pb_2_Br_6_, [Ni(bipy)_3_]AgBiI_6_, [Zn(bipy)_3_]_2_Ag_2_BiI_6_(I)_1.355_(I_3_)_1.645_ and [Co(bipy)_3_]_2_Ag_4_Bi_2_I_16_ [18,19,20,21,29,42,43].

## 3. Materials and Methods

### 3.1. General Information

Silver iodide (AgI, Adamas, 98%), lead iodide (PbI_2_, Aladdin, 98%), cobalt chloride hexahydrate (CoCl_2_·6H_2_O, Sinopharm, 99.9%), nickel chloride hexahydrate (NiCl_2_·6H_2_O, Sinopharm, 99.9%), potassium iodide (KI, Greagent, 99%), 1,10-phenanthroline (phen, Aladdin, 99%), 5,5-dimethyl-2,2-dipyridine (5,5-dmpy, Adamas, 98%), acetonitrile (CH_3_CN, Kermel, 99.5%) and hydriodic acid (HI, Adamas, 55–57 wt %). All the starting chemicals in this study were commercially available and need not be further disposal when used.

Purity identifications of samples were conducted by a SmartLab diffractometer. Thermogravimeric curves of title compounds were obtained using a NETZSCH STA449C unit (N_2_ atmosphere, 10 K/min). A Thermo Fisher GX4 scanning electron microscope and a 3600 SHIMADZU spectrometer were utilized to acquire the energy-dispersive X-ray (EDX) spectra and the solid optical diffuse reflectance data, respectively.

### 3.2. Syntheses

Synthesis of [Co(phen)_3_]Ag_2_PbI_6_ (**1**). The mixtures of PbI_2_ (0.332 g, 0.7 mmol), AgI (0.187 g, 0.8 mmol), KI (0.332 g, 2 mmol), CoCl_2_·6H_2_O (0.118 g, 0.5 mmol), phen (0.297 g, 1.5 mmol), hydroiodic acid (55–57 wt.%, 1 mL) and acetonitrile (99.5%, 4 mL) were placed in a 23 mL polytetrafluoroethylene-lined stainless steel autoclave. Subsequently, the temperature of resulting mixtures was maintained at 140 °C for 5 days. After the cooling and washing treatment, brown blocky crystals of **1** were harvested (38% yield based on PbI_2_). Elemental analysis calcd (%) for C_36_H_24_Ag_2_CoI_6_N_6_Pb (**1**): C 24.24, H 1.36, N 4.71; found: C 23.66, H 1.20, N 4.57.

Synthesis of [Ni(5,5-dmpy)_3_]Ag_7_I_9_·CH_3_CN (**2**). Compound **2** was also prepared by a typical solvothermal method. In detail, the chemicals including AgI (0.4108 g, 1.75 mmol), KI (0.2905 g, 1.75 mmol), NiCl_2_·6H_2_O (0.0594 g, 0.25 mmol), 5,5-dmpy (0.138 g, 0.75 mmol), hydroiodic acid (55–57 wt.%, 1 mL) and acetonitrile (99.5%, 4 mL) were added into a 23 mL polytetrafluoroethylene-lined container, which was kept at 140 °C for 5 days. After the filtration and the ethanol washing, pale-red block-like crystals were obtained by manual separation (yield: 36%, based on AgI). Elemental analysis calcd (%) for C_38_H_39_Ag_7_I_9_N_7_Ni (**2**): C 17.90, H 1.54, N 3.85; found: C 16.95, H 1.50, N 3.70.

**Scheme 1:**
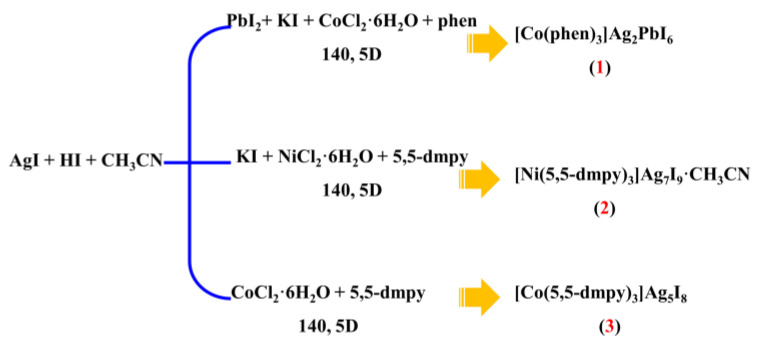
The summary of solvothermal preparations of compounds **1**–**3**.

Synthesis of [Co(5,5-dmpy)_3_]Ag_5_I_8_ (**3**). Compound **3** has the same synthesis process as compound **2**, but with different materials and dosages. Specifically, the reagents of AgI (0.3228 g, 1.375 mmol), CoCl_2_·6H_2_O (0.0595 g, 0.25 mmol), 5,5-dmpy (0.138 g, 0.75 mmol), hydroiodic acid (55–57 wt.%, 1 mL) and acetonitrile (99.5%, 4 mL) were put in a 23 mL polytetrafluoroethylene-lined stainless steel autoclave. Then, the mixture was heated at 140 °C for 5 days, followed by the gradual cooling to room temperature. Using a similar processing method mentioned above, black block-typed crystals of **3** were found, with the yield of 23% based on CoCl_2_·6H_2_O. Elemental analysis calcd (%) for C_36_H_36_Ag_5_CoI_8_N_6_ (**3**): C 19.96, H 1.68, N 3.88; found: C 19.92, H 1.61, N 3.76. The solvothermal syntheses of title compounds are summarized in Scheme 1.

### 3.3. X-ray Crystallography

Intensity data of title compounds were gathered on a Bruker SMART diffractometer (**1**, **3**) with an APEX II CCD detector and an Xcalibur E Oxford diffractometer (**2**) with an Atlas CCD detector using the graphite monochromatic Mo-K*α* radiation (*λ* = 0.71073 Å). Their structures were analyzed by a direct method and optimized on *F*^2^ by full-matrix least-squares technique using the SHELXTL–2014 program [44]. All non-hydrogen atoms were arranged anisotropically, and the hydrogen linked to carbon atoms were presented geometrically and refined isotropically under a fixed thermal factor. Their crystal data and structure refinement details are summarized in Table 1. The selected bond lengths and angles, hydrogen bond data, C–H⋯*π* interactions and *π*⋯*π* interactions are listed in Tables S1–S9.

### 3.4. Photocurrent Measurements

The photocurrent experiments of title compounds were carried out on a CHI 660E electrochemical workstation (Shanghai Chenhua Instrument Co., Ltd., Shanghai, China). In this study, we used a typical three-electrode configuration (the sample-coated ITO glass was the working electrode, the platinum wire was the counter electrode, and Ag/AgCl was the reference electrode). The sample/ITO electrode was prepared by the solution coating method. First, 5 mg of powder samples were added into a mixed solvent containing 475 µL ethanol and 25 µL Nafion. Ultrasonic treatment was carried out for 2 h, and the obtained solution was dropped on the surface of the pre-polished ITO glass, and then dried at room temperature. A 300-W Xenon lamp equipped with/without a 420-nm cut-off filter was used for the visible light and full spectrum light source. The electrolyte solution is the KCl solution (0.1 M).

### 3.5. Calculation Details

The calculation results including band structures, DOS and PDOS were obtained by density functional theory (DFT) using the VASP program. The exchange correlation function adopts the Perdew Burke Ernzerhof (PBE) function of the Generalized Gradient Approximation (GGA) [40,41]. Herein, H–1s^1^, C–2s^2^2p^2^, N–2s^2^2p^3^, Co–3d^7^4s^2^, Ni–3d^8^4s^2^, Ag–4d^10^5s^1^, Pb–6s^2^6p^2^ and I–5s^2^5p^5^ were served as the valence electrons. A cutoff energy of 500 eV was applied to define the number of plane waves. The numerical integration of the Brillouin zone was performed using Monkhorst-Pack *k*–point sampling of 3 × 3 × 3 for **1**, 3 × 5 × 3 for **2** and 1 × 2 × 2 for **3**, respectively.

## 4. Conclusions

In summary, the solvothermal syntheses, crystal structures, optical/photoelectric properties and theoretical studies of three new iodoargentate-based hybrids with metal complex cations were reported here. Compounds **1**–**3** possess chain-like structures, exhibiting semiconductor behaviors with the band gaps of 1.59–2.78 eV. Furthermore, the title compounds display interesting photoelectric switching performances upon the alternate light irradiations. Further work will focus on the fabrications of more metal complex-directed halide analogues and the in-depth understandings of their structure–activity relationships.

## Figures and Tables

**Table 1:** Crystallographic data of compounds **1**–**3**.

	1	2	3
formula	C_36_H_24_Ag_2_CoI_6_N_6_Pb	C_38_H_39_Ag_7_I_9_N_7_Ni	C_36_H_36_Ag_5_CoI_8_N_6_
fw	1783.87	2549.66	2166.19
crystal system	triclinic	monoclinic	monoclinic
space group	*P*-1 (No. 2)	*P*2_1_/*n* (No. 14)	*P*2/*c* (No. 13)
*a*/Ǻ	12.0376(11)	17.9597(8)	22.867(2)
*b*/Ǻ	13.8472(12)	13.1590(5)	13.4056(14)
*c*/Ǻ	14.8853(13)	24.1497(11)	17.2141(18)
*α*/°	112.785(4)	90	90
*β*/°	94.948(2)	101.825(4)	100.819(4)
*γ*/°	101.542(3)	90	90
*V*/Å^3^	2204.2(3)	5586.2(4)	5183.0(9)
*Z*	2	4	4
*T*/K	298(2)	293(2)	293(2)
*λ*/Ǻ	0.71073	0.71073	0.71073
*ρ*_calcd_/g cm^−3^	2.688	3.032	2.776
*F*(000)	1606	4600	3920
*R*_int_	0.0465	0.0396	0.1065
*GOF*	1.091	1.032	1.029
*R*_1_ [*I* > 2*s*(*I*)]	0.0725	0.0315	0.0776
*wR*(*F*_2_) [*I* > 2*s*(*I*)]	0.2053	0.0667	0.1942
CCDC	2,282,554	2,282,555	2,282,556

## Data Availability

Not applicable.

## Supplementary Materials

The following supporting information can be downloaded at: https://www.mdpi.com/article/10.3390/molecules28166116/s1, Table S1: Selected bond lengths (Å) and bond angles (°) for compound **1**; Table S2: Hydrogen bonds (Å) and angles (°) for compound **1**; Table S3: *π*⋯*π* interactions (Å and °) for compound **1**; Table S4 C–H⋯*π* interactions (Å and °) for compound **1**; Table S5: Selected bond lengths (Å) and bond angles (°) for compound **2**; Table S6: Hydrogen bonds (Å) and angles (°) for compound **2**; Table S7: C–H⋯*π* interactions (Å and °) for compound **2**; Table S8: Selected bond lengths (Å) and bond angles (°) for compound **3**; Table S9: Hydrogen bonds (Å) and angles (°) for compound **3**; Table S10: The Hirshfeld surface comparisons of compounds **1**–**3** with some related analogues; Table S11: The structural comparisons of compounds **1**–**3** with some related analogues. Figure S1: The [Ag_2_I_4_]*_n_*^2*n−*^ chain in compound **1**; Figure S2: The [Ag_4_I_9_] unit (a), [Ag_7_I_13_] unit (b) and [Ag_14_I_22_] unit (c) in compound **2**; Figure S3: The asymmetric unit of compound **3**; Figure S4: The [Ag_5_I_9_] unit (a) and [Ag_5_I_10_] unit (b) in compound **3**; Figure S5: Fingerprint plots: resolved into C⋯H (a), C⋯I (b) and C⋯C (c) for compound **1**; resolved into C⋯H (d), C⋯I (e) and C⋯C (f) for compound **2**; resolved into C⋯H (g), C⋯I (h) and Ag⋯Ag (i) for compound **3**; Figure S6: Experimental and simulated PXRD patterns of compound **1**; Figure S7: Experimental and simulated PXRD patterns of compound **2**; Figure S8: Experimental and simulated PXRD patterns of compound **3**; Figure S9: EDX spectrum of compound **1**; Figure S10: EDX spectrum of compound **2**; Figure S11: EDX spectrum of compound **3**; Figure S12: TGA curve of compound **1**; Figure S13: TGA curve of compound **2**; Figure S14: TGA curve of compound **3**; Figure S15: Total density of states and partial density of states for compound **1**. The Fermi level is set at 0 eV (dotted line); Figure S16: Total density of states and partial density of states for compound **2**. The Fermi level is set at 0 eV (dotted line); Figure S17: Total density of states and partial density of states for compound **3**. The Fermi level is set at 0 eV (dotted line).
