# Supplementary material for: Three Iodoargentate-Based Hybrids Decorated by Metal Complexes: Structures, Optical/Photoelectric Properties and Theoretical Studies
Source: Molecules. 2023 Aug 18;28(16):6116. doi: 10.3390/molecules28166116 (PMC10458760; doi:10.3390/molecules28166116)

checkCIF/PLATON report

You have not supplied any structure factors. As a result the full set of tests cannot be run.

THIS REPORT IS FOR GUIDANCE ONLY. IF USED AS PART OF A REVIEW PROCEDURE FOR PUBLICATION, IT SHOULD NOT REPLACE THE EXPERTISE OF AN EXPERIENCED CRYSTALLOGRAPHIC REFEREE.

No syntax errors found. CIF dictionary Interpreting this report

Datablock: Com-1

```
Bond precision:   C-C = 0.0300 A          Wavelength=0.71073

Cell:           a=12.0376(11)      b=13.8472(12)     c=14.8853(13)
                alpha=112.785(4)   beta=94.948(2)    gamma=101.542(3)
Temperature:    298 K

                 Calculated                   Reported
Volume           2204.2(3)                    2204.2(3)
Space group      P -1                         P -1
Hall group       -P 1                         -P 1
Moiety formula   Ag2 I6 Pb, C36 H24 Co N6     ?
Sum formula      C36 H24 Ag2 Co I6 N6 Pb      C36 H24 Ag2 Co I6 N6 Pb
Mr               1783.88                      1783.87
Dx,g cm-3        2.688                        2.688
Z                2                            2
Mu (mm-1)        9.287                        9.288
F000             1606.0                       1606.0
F000'            1591.44
h,k,lmax         14,16,17                     14,16,17
Nref             7790                         7638
Tmin,Tmax
Tmin'

Correction method= Not given

Data completeness= 0.980          Theta(max)= 25.017

R(reflections)= 0.0725( 5185)                        wR2(reflections)=
                                                     0.2222( 7638)
S = 1.091                  Npar= 469
```

The following ALERTS were generated. Each ALERT has the format
test-name_ALERT_alert-type_alert-level.
Click on the hyperlinks for more details of the test.

Alert level B

```
PLAT342_ALERT_3_B Low Bond Precision on  C-C Bonds ...............        0.03 Ang.
```

Alert level C

```
PLAT052_ALERT_1_C Info on Absorption Correction Method   Not Given     Please Do !
PLAT053_ALERT_1_C Minimum Crystal Dimension Missing (or Error) ...     Please Check
PLAT054_ALERT_1_C Medium  Crystal Dimension Missing (or Error) ...     Please Check
PLAT055_ALERT_1_C Maximum Crystal Dimension Missing (or Error) ...     Please Check
PLAT213_ALERT_2_C Atom C34             has ADP max/min Ratio .....       3.7 oblate
PLAT234_ALERT_4_C Large Hirshfeld Difference N2       --C6       .      0.18 Ang.
PLAT234_ALERT_4_C Large Hirshfeld Difference C8       --C9       .      0.19 Ang.
PLAT234_ALERT_4_C Large Hirshfeld Difference C22      --C23      .      0.24 Ang.
PLAT234_ALERT_4_C Large Hirshfeld Difference C25      --C26      .      0.21 Ang.
PLAT234_ALERT_4_C Large Hirshfeld Difference C29      --C33      .      0.17 Ang.
PLAT241_ALERT_2_C High   'MainMol' Ueq as Compared to Neighbors of        C8 Check
PLAT241_ALERT_2_C High   'MainMol' Ueq as Compared to Neighbors of       C18 Check
PLAT241_ALERT_2_C High   'MainMol' Ueq as Compared to Neighbors of       C23 Check
PLAT243_ALERT_4_C High   'Solvent' Ueq as Compared to Neighbors of       Ag1 Check
PLAT244_ALERT_4_C Low    'Solvent' Ueq as Compared to Neighbors of        I4 Check
PLAT244_ALERT_4_C Low    'Solvent' Ueq as Compared to Neighbors of        I5 Check
PLAT250_ALERT_2_C Large U3/U1 Ratio for Average U(i,j) Tensor ....       2.4 Note
```

Alert level G

```
PLAT004_ALERT_5_G Polymeric Structure Found with Maximum Dimension          1 Info
PLAT083_ALERT_2_G SHELXL Second Parameter in WGHT  Unusually Large       7.06 Why ?
PLAT233_ALERT_4_G Hirshfeld (M-X Solvent)    Pb1      --I1       .        6.9 s.u.
PLAT233_ALERT_4_G Hirshfeld (M-X Solvent)    Pb1      --I3       .       16.6 s.u.
PLAT233_ALERT_4_G Hirshfeld (M-X Solvent)    Pb1      --I4       .        8.0 s.u.
PLAT233_ALERT_4_G Hirshfeld (M-X Solvent)    Pb1      --I6_b     .        7.7 s.u.
PLAT233_ALERT_4_G Hirshfeld (M-X Solvent)    Pb1      --I5_d     .        8.3 s.u.
PLAT233_ALERT_4_G Hirshfeld (M-X Solvent)    I1       --Ag2_d    .       12.6 s.u.
PLAT233_ALERT_4_G Hirshfeld (M-X Solvent)    I4       --Ag1      .        7.5 s.u.
PLAT233_ALERT_4_G Hirshfeld (M-X Solvent)    I4       --Ag1_d    .       26.0 s.u.
PLAT233_ALERT_4_G Hirshfeld (M-X Solvent)    I5       --Ag1      .        8.0 s.u.
PLAT233_ALERT_4_G Hirshfeld (M-X Solvent)    I5       --Ag2      .       19.3 s.u.
PLAT233_ALERT_4_G Hirshfeld (M-X Solvent)    I6       --Ag2      .        5.6 s.u.
PLAT233_ALERT_4_G Hirshfeld (M-X Solvent)    I6       --Ag1_c    .       96.0 s.u.
PLAT233_ALERT_4_G Hirshfeld (M-X Solvent)    I6       --Ag2_c    .       25.4 s.u.
PLAT333_ALERT_2_G Large Aver C6-Ring C-C Dist C4       -C8       .       1.43 Ang.
PLAT335_ALERT_2_G Check Large C6 Ring C-C Range C4       -C8              0.17 Ang.
PLAT335_ALERT_2_G Check Large C6 Ring C-C Range C16      -C18             0.23 Ang.
PLAT335_ALERT_2_G Check Large C6 Ring C-C Range C28      -C30             0.16 Ang.
PLAT480_ALERT_4_G Long H...A H-Bond Reported H13      ..I2       .       3.27 Ang.
PLAT480_ALERT_4_G Long H...A H-Bond Reported H23      ..I5       .       3.27 Ang.
PLAT480_ALERT_4_G Long H...A H-Bond Reported H35      ..I1       .       3.31 Ang.
PLAT794_ALERT_5_G Tentative Bond Valency for Pb1       (II)      .       2.08 Info
PLAT794_ALERT_5_G Tentative Bond Valency for Co1       (II)      .       1.82 Info
PLAT883_ALERT_1_G No Info/Value for _atom_sites_solution_primary .     Please Do !
```

```
PLAT941_ALERT_3_G Average HKL Measurement Multiplicity ...........       1.4 Low
PLAT965_ALERT_2_G The SHELXL WEIGHT Optimisation has not Converged     Please Check
PLAT967_ALERT_5_G Note: Two-Theta Cutoff Value in Embedded .res ..      50.0 Degree
```

```
   0 ALERT level A = Most likely a serious problem - resolve or explain
   1 ALERT level B = A potentially serious problem, consider carefully
  17 ALERT level C = Check. Ensure it is not caused by an omission or oversight
  28 ALERT level G = General information/check it is not something unexpected

   5 ALERT type 1 CIF construction/syntax error, inconsistent or missing data
  11 ALERT type 2 Indicator that the structure model may be wrong or deficient
   2 ALERT type 3 Indicator that the structure quality may be low
  24 ALERT type 4 Improvement, methodology, query or suggestion
   4 ALERT type 5 Informative message, check
```

Datablock: Com-2

Bond precision: C-C = 0.0092 A Wavelength=0.71073

Cell:	a=17.9597(8)	b=13.1590(5)	c=24.1497(11)
	alpha=90	beta=101.825(4)	gamma=90
Temperature:	293 K		

	Calculated	Reported
Volume	5586.2(4)	5586.2(4)
Space group	P 21/n	P 21/n
Hall group	-P 2yn	-P 2yn
Moiety formula	Ag7 I9, C36 H36 N6 Ni, C2 H3 N	?
Sum formula	C38 H39 Ag7 I9 N7 Ni	C38 H39 Ag7 I9 N7 Ni
Mr	2549.64	2549.66
Dx,g cm-3	3.032	3.032
Z	4	4
Mu (mm-1)	7.738	7.738
F000	4600.0	4600.0
F000'	4560.62	
h,k,lmax	22,16,29	22,15,29
Nref	10971	10735
Tmin,Tmax		
Tmin'		

Correction method= Not given

Data completeness= 0.978 Theta(max)= 25.996

R(reflections)= 0.0315(9270)

wR2(reflections)= 0.0703(10735)

S = 1.032 Npar= 576

The following ALERTS were generated. Each ALERT has the format
test-name_ALERT_alert-type_alert-level.
Click on the hyperlinks for more details of the test.

Alert level C

```
PLAT052_ALERT_1_C Info on Absorption Correction Method   Not Given     Please Do !
PLAT053_ALERT_1_C Minimum Crystal Dimension Missing (or Error) ...     Please Check
PLAT054_ALERT_1_C Medium  Crystal Dimension Missing (or Error) ...     Please Check
PLAT055_ALERT_1_C Maximum Crystal Dimension Missing (or Error) ...     Please Check
PLAT213_ALERT_2_C Atom Ag7A            has ADP max/min Ratio .....        3.1 prolat
PLAT342_ALERT_3_C Low Bond Precision on  C-C Bonds ..............     0.00921 Ang.
```

Alert level G

```
PLAT004_ALERT_5_G Polymeric Structure Found with Maximum Dimension          1 Info
PLAT083_ALERT_2_G SHELXL Second Parameter in WGHT  Unusually Large       6.23 Why ?
PLAT199_ALERT_1_G Reported _cell_measurement_temperature ..... (K)        293 Check
PLAT200_ALERT_1_G Reported   _diffrn_ambient_temperature ..... (K)        293 Check
PLAT232_ALERT_2_G Hirshfeld Test Diff (M-X)  I1       --Ag1      .        7.2 s.u.
PLAT232_ALERT_2_G Hirshfeld Test Diff (M-X)  I2       --Ag1      .        8.2 s.u.
PLAT232_ALERT_2_G Hirshfeld Test Diff (M-X)  I2       --Ag2      .       18.0 s.u.
PLAT232_ALERT_2_G Hirshfeld Test Diff (M-X)  I2       --Ag5      .       10.8 s.u.
PLAT232_ALERT_2_G Hirshfeld Test Diff (M-X)  I3       --Ag2      .        9.7 s.u.
PLAT232_ALERT_2_G Hirshfeld Test Diff (M-X)  I3       --Ag4_a    .       12.2 s.u.
PLAT232_ALERT_2_G Hirshfeld Test Diff (M-X)  I4       --Ag1      .       35.0 s.u.
PLAT232_ALERT_2_G Hirshfeld Test Diff (M-X)  I4       --Ag2      .       37.0 s.u.
PLAT232_ALERT_2_G Hirshfeld Test Diff (M-X)  I4       --Ag3      .       20.3 s.u.
PLAT232_ALERT_2_G Hirshfeld Test Diff (M-X)  I4       --Ag7      .       40.5 s.u.
PLAT232_ALERT_2_G Hirshfeld Test Diff (M-X)  I4       --Ag6_a    .       24.5 s.u.
PLAT232_ALERT_2_G Hirshfeld Test Diff (M-X)  I4       --Ag7_a    .       40.5 s.u.
PLAT232_ALERT_2_G Hirshfeld Test Diff (M-X)  I5       --Ag2      .       14.0 s.u.
PLAT232_ALERT_2_G Hirshfeld Test Diff (M-X)  I6       --Ag3      .       10.2 s.u.
PLAT232_ALERT_2_G Hirshfeld Test Diff (M-X)  I6       --Ag4      .       15.2 s.u.
PLAT232_ALERT_2_G Hirshfeld Test Diff (M-X)  I6       --Ag6_a    .       14.8 s.u.
PLAT232_ALERT_2_G Hirshfeld Test Diff (M-X)  I7       --Ag3      .       25.5 s.u.
PLAT232_ALERT_2_G Hirshfeld Test Diff (M-X)  I7       --Ag4      .       28.8 s.u.
PLAT232_ALERT_2_G Hirshfeld Test Diff (M-X)  I7       --Ag5      .        9.8 s.u.
PLAT232_ALERT_2_G Hirshfeld Test Diff (M-X)  I7       --Ag7A     .        6.0 s.u.
PLAT232_ALERT_2_G Hirshfeld Test Diff (M-X)  I8       --Ag1      .       21.3 s.u.
PLAT232_ALERT_2_G Hirshfeld Test Diff (M-X)  I8       --Ag5      .        7.0 s.u.
PLAT232_ALERT_2_G Hirshfeld Test Diff (M-X)  I8       --Ag6      .       77.8 s.u.
PLAT232_ALERT_2_G Hirshfeld Test Diff (M-X)  I9       --Ag5      .        7.2 s.u.
PLAT232_ALERT_2_G Hirshfeld Test Diff (M-X)  I9       --Ag6      .        6.5 s.u.
PLAT301_ALERT_3_G Main Residue  Disorder .............(Resd  1  )         6% Note
PLAT480_ALERT_4_G Long H...A H-Bond Reported H1       ..I6       .       3.23 Ang.
PLAT480_ALERT_4_G Long H...A H-Bond Reported H8       ..I6       .       3.13 Ang.
PLAT480_ALERT_4_G Long H...A H-Bond Reported H12      ..N4       .       2.63 Ang.
PLAT480_ALERT_4_G Long H...A H-Bond Reported H20      ..I9       .       3.30 Ang.
PLAT480_ALERT_4_G Long H...A H-Bond Reported H32      ..I5       .       3.26 Ang.
PLAT480_ALERT_4_G Long H...A H-Bond Reported H36A     ..I9       .       3.14 Ang.
```

```
PLAT480_ALERT_4_G Long H...A H-Bond Reported H36B     ..I5       .       3.12 Ang.
PLAT480_ALERT_4_G Long H...A H-Bond Reported H36C     ..I7       .       3.29 Ang.
PLAT794_ALERT_5_G Tentative Bond Valency for Ni1       (II)      .       2.16 Info
PLAT883_ALERT_1_G No Info/Value for _atom_sites_solution_primary .     Please Do !
PLAT941_ALERT_3_G Average HKL Measurement Multiplicity ..........        2.6 Low
PLAT965_ALERT_2_G The SHELXL WEIGHT Optimisation has not Converged     Please Check
PLAT967_ALERT_5_G Note: Two-Theta Cutoff Value in Embedded .res ..       52.0 Degree
```

```
   0 ALERT level A = Most likely a serious problem - resolve or explain
   0 ALERT level B = A potentially serious problem, consider carefully
   6 ALERT level C = Check. Ensure it is not caused by an omission or oversight
  43 ALERT level G = General information/check it is not something unexpected

   7 ALERT type 1 CIF construction/syntax error, inconsistent or missing data
  28 ALERT type 2 Indicator that the structure model may be wrong or deficient
   3 ALERT type 3 Indicator that the structure quality may be low
   8 ALERT type 4 Improvement, methodology, query or suggestion
   3 ALERT type 5 Informative message, check
```

Datablock: Com-3

Bond precision:	C-C = 0.0318 A		Wavelength=0.71073
Cell:	a=22.867(2)	b=13.4056(14)	c=17.2141(18)
	alpha=90	beta=100.819(4)	gamma=90
Temperature:	293 K		

	Calculated	Reported
Volume	5183.1(9)	5183.0(9)
Space group	P 2/c	P 2/c
Hall group	-P 2yc	-P 2yc
Moiety formula	C36 H36 Co N6, 0.5(Ag5 I8), Ag2.50 I4	?
Sum formula	C36 H36 Ag5 Co I8 N6	C36 H36 Ag5 Co I8 N6
Mr	2166.19	2166.19
Dx,g cm-3	2.776	2.776
Z	4	4
Mu (mm-1)	6.961	6.962
F000	3920.0	3920.0
F000'	3889.52	
h,k,lmax	27,15,20	27,15,20
Nref	9143	9118
Tmin,Tmax		
Tmin'		

Correction method= Not given

Data completeness= 0.997 Theta(max)= 25.019

R(reflections)= 0.0776(4109) wR2(reflections)= 0.2346(9118)

S = 1.029 Npar= 528

The following ALERTS were generated. Each ALERT has the format
test-name_ALERT_alert-type_alert-level.
Click on the hyperlinks for more details of the test.

Alert level B

```
PLAT342_ALERT_3_B Low Bond Precision on  C-C Bonds ..............    0.03182 Ang.
```

Alert level C

```
PLAT026_ALERT_3_C Ratio Observed / Unique Reflections (too) Low ..       45% Check
PLAT052_ALERT_1_C Info on Absorption Correction Method   Not Given     Please Do !
PLAT053_ALERT_1_C Minimum Crystal Dimension Missing (or Error) ...     Please Check
PLAT054_ALERT_1_C Medium  Crystal Dimension Missing (or Error) ...     Please Check
PLAT055_ALERT_1_C Maximum Crystal Dimension Missing (or Error) ...     Please Check
PLAT085_ALERT_2_C SHELXL Default Weighting Scheme is not Optimized     Please Check
PLAT213_ALERT_2_C Atom N4              has ADP max/min Ratio .....      3.7 prolat
PLAT213_ALERT_2_C Atom N6              has ADP max/min Ratio .....      4.0 oblate
PLAT213_ALERT_2_C Atom C4              has ADP max/min Ratio .....      3.6 prolat
PLAT213_ALERT_2_C Atom C6              has ADP max/min Ratio .....      3.1 oblate
PLAT213_ALERT_2_C Atom C16             has ADP max/min Ratio .....      3.4 oblate
PLAT220_ALERT_2_C NonSolvent   Resd 1  C    Ueq(max)/Ueq(min) Range     3.8 Ratio
PLAT222_ALERT_3_C NonSolvent Resd 1  H   Uiso(max)/Uiso(min) Range      4.7 Ratio
PLAT234_ALERT_4_C Large Hirshfeld Difference N1      --C6       .      0.18 Ang.
PLAT234_ALERT_4_C Large Hirshfeld Difference N2      --C7       .      0.20 Ang.
PLAT234_ALERT_4_C Large Hirshfeld Difference N3      --C13      .      0.21 Ang.
PLAT234_ALERT_4_C Large Hirshfeld Difference C14     --C16      .      0.24 Ang.
PLAT234_ALERT_4_C Large Hirshfeld Difference I4A     --Ag3      .      0.17 Ang.
PLAT241_ALERT_2_C High   'MainMol' Ueq as Compared to Neighbors of      C16 Check
PLAT241_ALERT_2_C High   'MainMol' Ueq as Compared to Neighbors of      C20 Check
PLAT241_ALERT_2_C High   'MainMol' Ueq as Compared to Neighbors of      C28 Check
PLAT241_ALERT_2_C High   'MainMol' Ueq as Compared to Neighbors of      Ag3 Check
PLAT242_ALERT_2_C Low    'MainMol' Ueq as Compared to Neighbors of      C23 Check
PLAT242_ALERT_2_C Low    'MainMol' Ueq as Compared to Neighbors of       I2 Check
PLAT243_ALERT_4_C High   'Solvent' Ueq as Compared to Neighbors of      Ag4 Check
PLAT243_ALERT_4_C High   'Solvent' Ueq as Compared to Neighbors of      Ag5 Check
PLAT244_ALERT_4_C Low    'Solvent' Ueq as Compared to Neighbors of       I7 Check
PLAT244_ALERT_4_C Low    'Solvent' Ueq as Compared to Neighbors of       I8 Check
PLAT244_ALERT_4_C Low    'Solvent' Ueq as Compared to Neighbors of       I9 Check
```

Alert level G

```
PLAT003_ALERT_2_G Number of Uiso or Uij Restrained non-H Atoms ...        9 Report
PLAT004_ALERT_5_G Polymeric Structure Found with Maximum Dimension        1 Info
PLAT177_ALERT_4_G The CIF-Embedded .res File Contains DELU Records        1 Report
```

```
PLAT186_ALERT_4_G The CIF-Embedded .res File Contains ISOR Records          2 Report
PLAT192_ALERT_3_G A Non-default DELU Restraint Value for First Par     0.0010 Report
PLAT199_ALERT_1_G Reported _cell_measurement_temperature ..... (K)        293 Check
PLAT200_ALERT_1_G Reported   _diffrn_ambient_temperature ..... (K)        293 Check
PLAT232_ALERT_2_G Hirshfeld Test Diff (M-X)  I1       --Ag2      .        7.1 s.u.
PLAT232_ALERT_2_G Hirshfeld Test Diff (M-X)  I2       --Ag1      .       30.0 s.u.
PLAT232_ALERT_2_G Hirshfeld Test Diff (M-X)  I2       --Ag2      .       24.0 s.u.
PLAT232_ALERT_2_G Hirshfeld Test Diff (M-X)  I2       --Ag3      .       29.3 s.u.
PLAT232_ALERT_2_G Hirshfeld Test Diff (M-X)  I2       --Ag2_a    .       17.6 s.u.
PLAT232_ALERT_2_G Hirshfeld Test Diff (M-X)  I3       --Ag3      .        9.4 s.u.
PLAT232_ALERT_2_G Hirshfeld Test Diff (M-X)  I5       --Ag3      .        8.4 s.u.
PLAT233_ALERT_4_G Hirshfeld (M-X Solvent)    I6       --Ag4      .        8.5 s.u.
PLAT233_ALERT_4_G Hirshfeld (M-X Solvent)    I6       --Ag4_e    .        9.5 s.u.
PLAT233_ALERT_4_G Hirshfeld (M-X Solvent)    I7       --Ag4      .       23.5 s.u.
PLAT233_ALERT_4_G Hirshfeld (M-X Solvent)    I7       --Ag5      .       37.0 s.u.
PLAT233_ALERT_4_G Hirshfeld (M-X Solvent)    I7       --Ag6      .        9.5 s.u.
PLAT233_ALERT_4_G Hirshfeld (M-X Solvent)    I7       --Ag5_d    .       22.0 s.u.
PLAT233_ALERT_4_G Hirshfeld (M-X Solvent)    I7       --Ag6_d    .       15.2 s.u.
PLAT233_ALERT_4_G Hirshfeld (M-X Solvent)    I9       --Ag5      .       35.5 s.u.
PLAT300_ALERT_4_G Atom Site Occupancy of Ag6       Constrained at         0.5 Check
PLAT302_ALERT_4_G Anion/Solvent/Minor-Residue Disorder (Resd  2  )        12% Note
PLAT302_ALERT_4_G Anion/Solvent/Minor-Residue Disorder (Resd  3  )         8% Note
PLAT480_ALERT_4_G Long H...A H-Bond Reported H5       ..I8       .       3.16 Ang.
PLAT480_ALERT_4_G Long H...A H-Bond Reported H8       ..I8       .       3.23 Ang.
PLAT480_ALERT_4_G Long H...A H-Bond Reported H17      ..I5       .       3.09 Ang.
PLAT779_ALERT_4_G Suspect or Irrelevant (Bond) Angle(s) in CIF ...      18.00 Deg.
              AG6  -I9   -AG6     1_555   1_555   2_755 ......     #   22 Check
PLAT794_ALERT_5_G Tentative Bond Valency for Co1       (III)     .       3.77 Info
PLAT860_ALERT_3_G Number of Least-Squares Restraints ............          43 Note
PLAT883_ALERT_1_G No Info/Value for _atom_sites_solution_primary .     Please Do !
PLAT933_ALERT_2_G Number of HKL-OMIT Records in Embedded .res File          2 Note
PLAT941_ALERT_3_G Average HKL Measurement Multiplicity ..........         2.6 Low
PLAT965_ALERT_2_G The SHELXL WEIGHT Optimisation has not Converged     Please Check
PLAT967_ALERT_5_G Note: Two-Theta Cutoff Value in Embedded .res ..       50.0 Degree
```

```
   0 ALERT level A = Most likely a serious problem - resolve or explain
   1 ALERT level B = A potentially serious problem, consider carefully
  29 ALERT level C = Check. Ensure it is not caused by an omission or oversight
  36 ALERT level G = General information/check it is not something unexpected

   7 ALERT type 1 CIF construction/syntax error, inconsistent or missing data
  23 ALERT type 2 Indicator that the structure model may be wrong or deficient
   6 ALERT type 3 Indicator that the structure quality may be low
  27 ALERT type 4 Improvement, methodology, query or suggestion
   3 ALERT type 5 Informative message, check
```

Validation response form

Please find below a validation response form (VRF) that can be filled in and pasted into your CIF.

```
# start Validation Reply Form
_vrf_PLAT342_Com-1
;
PROBLEM: Low Bond Precision on  C-C Bonds ..............       0.03 Ang.
RESPONSE: ...
```

```
;
_vrf_PLAT342_Com-3
;
PROBLEM: Low Bond Precision on  C-C Bonds ..............    0.03182 Ang.
RESPONSE: ...
;
# end Validation Reply Form
```

It is advisable to attempt to resolve as many as possible of the alerts in all categories. Often the minor alerts point to easily fixed oversights, errors and omissions in your CIF or refinement strategy, so attention to these fine details can be worthwhile. In order to resolve some of the more serious problems it may be necessary to carry out additional measurements or structure refinements. However, the purpose of your study may justify the reported deviations and the more serious of these should normally be commented upon in the discussion or experimental section of a paper or in the "special_details" fields of the CIF. checkCIF was carefully designed to identify outliers and unusual parameters, but every test has its limitations and alerts that are not important in a particular case may appear. Conversely, the absence of alerts does not guarantee there are no aspects of the results needing attention. It is up to the individual to critically assess their own results and, if necessary, seek expert advice.

Publication of your CIF in IUCr journals

A basic structural check has been run on your CIF. These basic checks will be run on all CIFs submitted for publication in IUCr journals (*Acta Crystallographica*, *Journal of Applied Crystallography*, *Journal of Synchrotron Radiation*); however, if you intend to submit to *Acta Crystallographica Section C* or *E* or *IUCrData*, you should make sure that full publication checks are run on the final version of your CIF prior to submission.

Publication of your CIF in other journals

Please refer to the *Notes for Authors* of the relevant journal for any special instructions relating to CIF submission.

PLATON version of 06/07/2023; check.def file version of 30/06/2023

Datablock Com-1 - ellipsoid plot

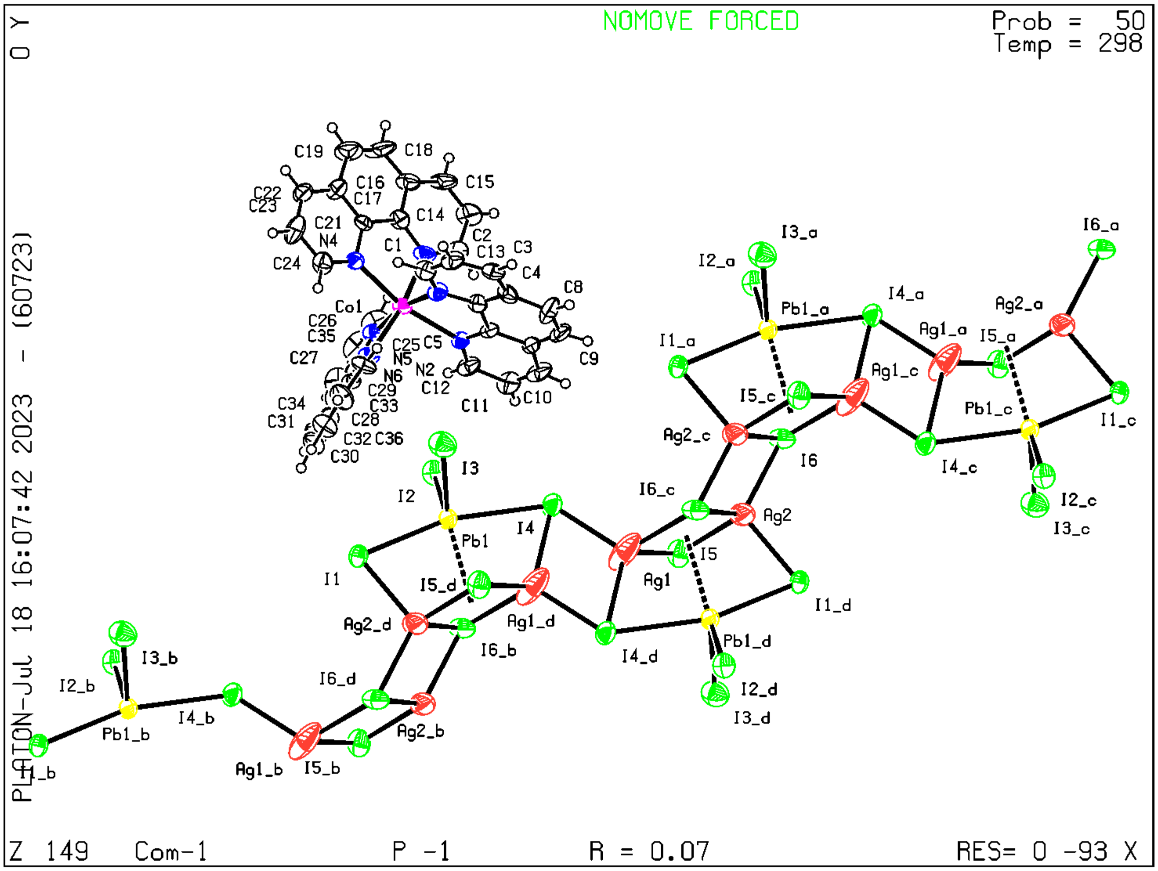

Datablock Com-2 - ellipsoid plot

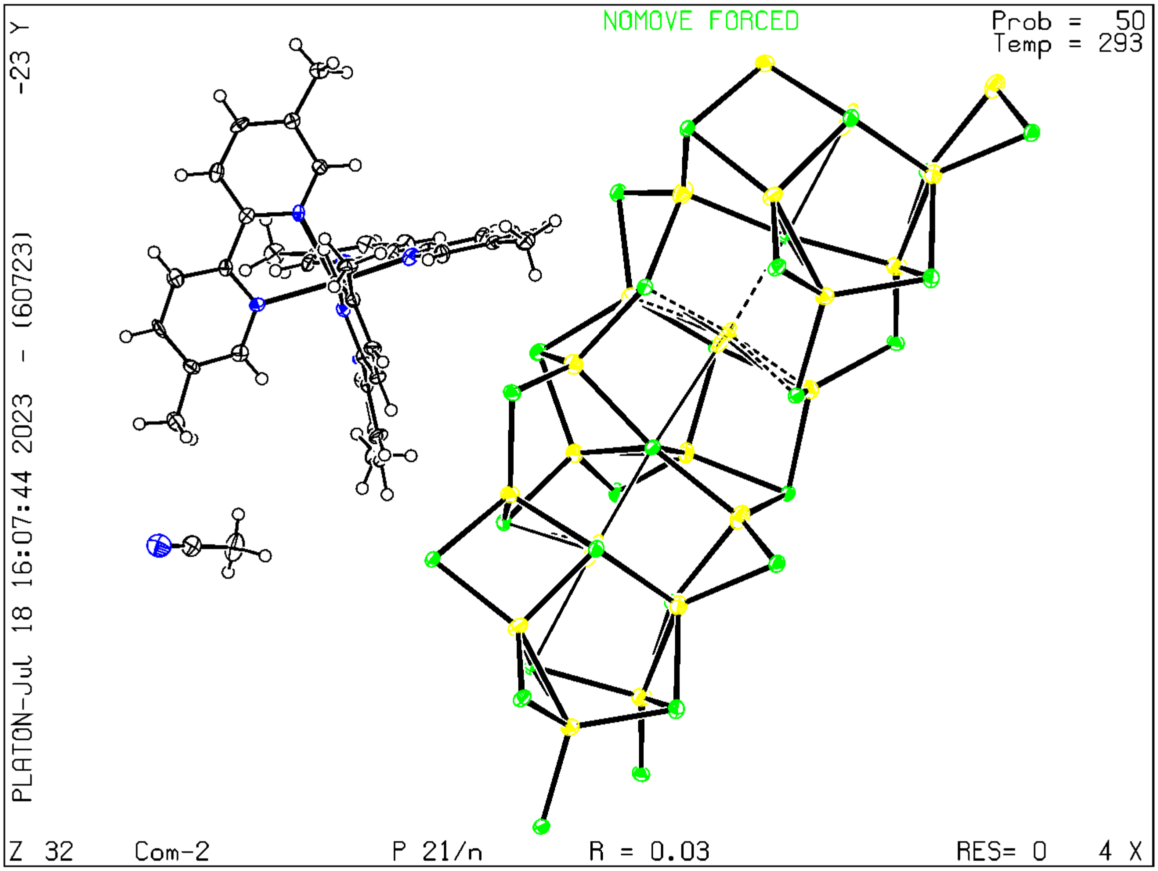

Datablock Com-3 - ellipsoid plot

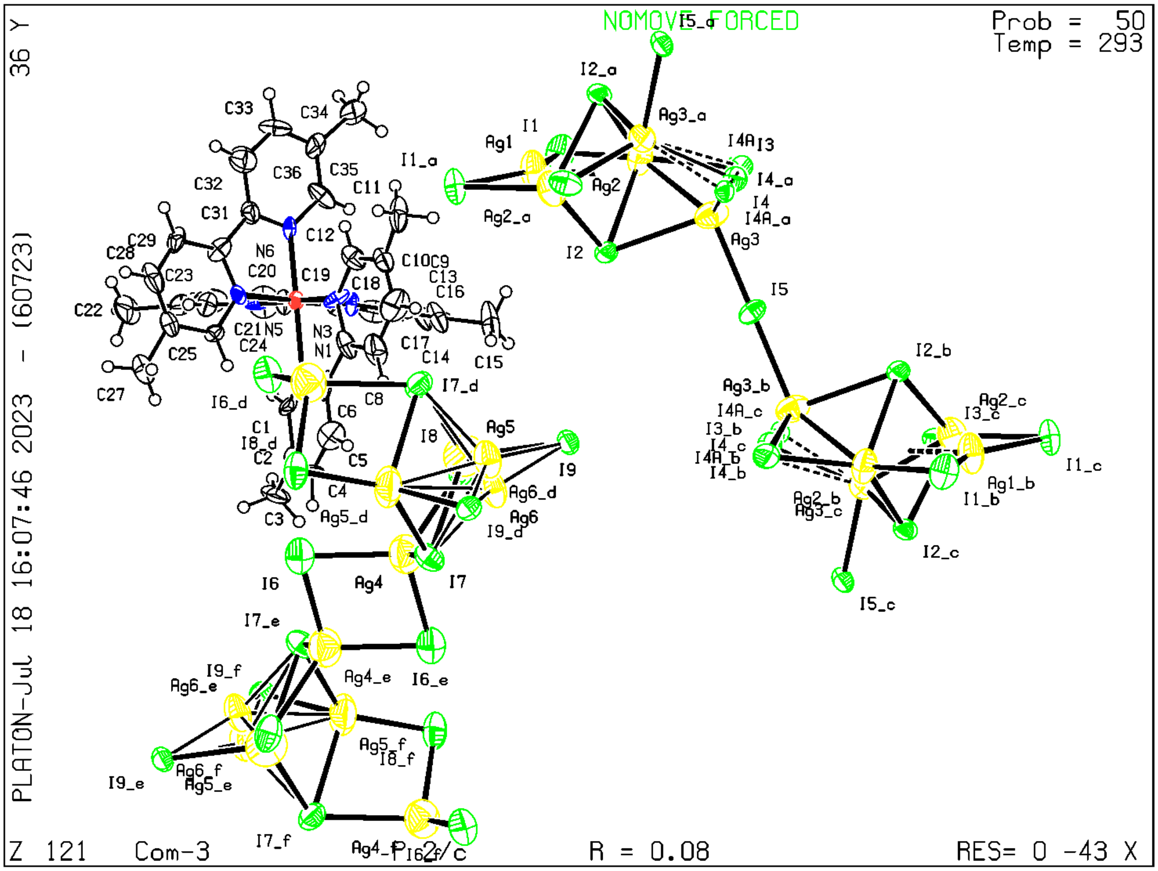

Supplement: Supplementary file 1 [file molecules-28-06116-s001.zip › checkcif.pdf]
