# Supplementary material for: Three Iodoargentate-Based Hybrids Decorated by Metal Complexes: Structures, Optical/Photoelectric Properties and Theoretical Studies
Source: Molecules. 2023 Aug 18;28(16):6116. doi: 10.3390/molecules28166116 (PMC10458760; doi:10.3390/molecules28166116)
Supplement: Supplementary file 1 [file molecules-28-06116-s001.zip › Supporting Information.pdf]

Supporting Information

Three Iodoargentate-Based Hybrids Decorated by Metal Complexes: Structures, Optical/Photoelectric Properties and Theoretical Studies

Minghui Liu[1†], Xiaochen Ren[1†], Weiyang Wen[2], Baohan Li[1], Jiaqi Li[1], Jun Li[1,*], and Bo Zhang[1,*]

1 Department of Chemistry and Chemical Engineering, Liaocheng University, Liaocheng 252059, China; mhliu_lcu@163.com (M.L.); renxiaochen777@163.com (X.R.); bhli_lcu@163.com (B.L.); jiaqili_lcu@163.com (J.L.)

2 State Key Laboratory of Structural Chemistry, Fujian Institute of Research on the Structure of Matter, Chinese Academy of Sciences, Fuzhou 350002, China; wenweiyang@fjirsm.ac.cn (W.Y.W.)

* Correspondence: junli@lcu.edu.cn (J.L.); bzhang@lcu.edu.cn (B.Z.)

† These authors contributed equally to this work.

Table of Contents

Tables S1−S9: Additional structural details of compounds **1**−**3**.

Figures S1−S4: Crystal structures of compounds **1**−**3**.

Figure S5: Hirshfeld surface analyses of compounds **1**−**3**.

Table S10: The Hirshfeld surface comparisons of compounds **1**−**3** with some related analogues.

Figures S6−S8: PXRD patterns of compounds **1**−**3**.

Figures S9−S11: EDX spectra of compounds **1**−**3**.

Figures S12−S14: TGA curves of compounds **1**−**3**.

Figures S15−S17: DFT calculations.

Table S11: The structural comparisons of compounds **1**−**3** with some related analogues.

Reference

1. Additional structural details

Table S1 Selected bond lengths (Å) and bond angles (°) for compound **1**.

Pb(1)−I(1)	3.2615(14)	Ag(2)−I(6)	2.847(2)
Pb(1)−I(2)	3.0390(15)	Ag(2)−I(6)#2	2.991(2)
Pb(1)−I(3)	2.8320(19)	Co(1)−N(1)	2.183(14)
Pb(1)−I(4)	3.4246(15)	Co(1)−N(2)	2.198(13)
Ag(1)−I(4)#1	2.938(3)	Co(1)−N(3)	2.155(13)
Ag(1)−I(4)	2.809(2)	Co(1)−N(4)	2.160(13)
Ag(1)−I(5)	2.780(2)	Co(1)−N(5)	2.145(15)
Ag(2)−I(1)#1	2.889(2)	Co(1)−N(6)	2.134(13)
Ag(2)−I(5)	2.914(3)		
I(1)−Pb(1)−I(4)	162.62(4)	N(1)−Co(1)−N(2)	76.9(5)
I(2)−Pb(1)−I(4)	89.32(4)	N(3)−Co(1)−N(1)	95.2(6)
I(2)−Pb(1)−I(1)	106.40(4)	N(3)−Co(1)−N(2)	92.1(5)
I(3)−Pb(1)−I(1)	96.81(4)	N(3)−Co(1)−N(4)	78.3(5)
I(3)−Pb(1)−I(2)	90.53(5)	N(4)−Co(1)−N(1)	94.4(5)
I(3)−Pb(1)−I(4)	90.25(5)	N(4)−Co(1)−N(2)	166.5(5)
I(4)−Ag(1)−I(4)#1	109.37(9)	N(5)−Co(1)−N(1)	167.7(5)
I(5)−Ag(1)−I(4)#1	114.46(10)	N(5)−Co(1)−N(2)	93.3(5)
I(5)−Ag(1)−I(4)	118.63(10)	N(5)−Co(1)−N(3)	92.5(5)
I(1)#1−Ag(2)−I(5)	101.47(7)	N(5)−Co(1)−N(4)	96.5(5)
I(1)#1−Ag(2)−I(6)#1	103.93(7)	N(6)−Co(1)−N(1)	94.3(5)
I(5)−Ag(2)−I(6)#2	98.14(7)	N(6)−Co(1)−N(2)	96.7(5)
I(6)−Ag(2)−I(1)#1	113.56(7)	N(6)−Co(1)−N(3)	168.3(6)
I(6)−Ag(2)−I(5)	124.21(8)	N(6)−Co(1)−N(4)	94.2(5)
I(6)−Ag(2)−I(6)#2	112.79(7)	N(6)−Co(1)−N(5)	79.3(5)

Symmetry transformations used to generate equivalent atoms: #1 −*x*+1, −*y*, −*z*; #2 −*x*, −*y*, −*z*.

Table S2 Hydrogen bonds (Å) and angles (º) for compound **1**.

C−H···I	*d*(C−H)	*d*(H···I)	*d*(C···I)	<(CHI)
C(8)−H(8)···I(3)	0.93	2.93	3.7968	155
C(13)−H(13)···I(2)#3	0.93	3.27	3.968(16)	133.8
C(23)−H(23)···I(5)#4	0.93	3.27	3.94(2)	129.9
C(30)−H(30)···I(2)#5	0.93	3.01	3.8462	151
C(36)−H(36)···I(3)#6	0.93	3.02	3.78(2)	139.9

Symmetry transformations used to generate equivalent atoms: #3 −*x*+1, −*y*, −*z*+1; #4 *x*+1, *y*+1, *z*+1; #5 *x*−1, *y*+1, *z*; #6 −*x*+2, −*y*+1, −*z*+1.

Table S3 $\pi\cdots\pi$ interactions (Å and °) for compound **1**.

Cg(i)···Cg(j)	*d*[Cg(i)···Cg(j)][a]	α^b	β^c	γ^d	*d*[Cg(i)···Perp][e]	*d*[Cg(j)···Perp][f]
Cg(1)···Cg(2)#7	3.852(12)	0.48	17.26	17.32	3.677	3.679

Symmetry transformations used to generate equivalent atoms: #7 −*x*+1, −*y*+1, −*z*.

Cg(1): N(3)−C(13)−C(14)−C(15)−C(16)−C(17); Cg(2): C(16)−C(17)−C(21)−C(20)−C(19)−C(18).

Table S4 C−H···π interactions (Å and ˚) for compound **1**.

C−H(I)···Cg	*d*(H···Cg)	*d*(C···Cg)	∠(C−H···Cg)
C(18)−H(18)···Cg(3)#8	2.67	3.590(2)	172
C(22)−H(22)···Cg(4)#8	2.69	3.579(3)	161

Symmetry codes: #8 −*x*, −*y*+2, 1−*z*. Cg(3): N(1)−C(1)−C(2)−C(3)−C(4)−C(5); Cg(4): C(29)−C(28)−C(30)−C(31)−C(32)−C(33).

Table S5 Selected bond lengths (Å) and bond angles (°) for compound **2**.

Ag(1)−I(2)	3.0850(2)	I(2)−Ag(2)	2.9693(17)
Ag(1)−I(4)#1	2.7820(19)	I(2)−Ag(3)	2.8882(18)
Ag(1)−I(5)#1	2.9150(2)	I(2)−Ag(4)	2.7645(18)
Ag(2)−I(3)	2.8693(18)	I(3)−Ag(4)	2.8441(19)
Ag(2)−I(6)	2.8462(18)	I(3)−Ag(5)	3.0130(2)
Ag(2)−Ag(3)	3.1850(2)	I(3)−Ag(6)	2.9030(2)
Ag(2)−Ag(4)	3.1290(2)	I(4)−Ag(1)#2	2.7819(19)
Ag(3)−I(5)	3.0056(19)	I(4)−Ag(5)	2.9171(19)
Ag(3)−I(6)	2.7767(18)	I(4)−Ag(6)	2.8421(19)
Ag(3)−I(7)	2.7223(17)	I(5)−Ag(1)#2	2.9150(2)
Ag(3)−Ag(7)	3.3540(2)	I(5)−Ag(6)	2.9392(18)
Ag(4)−I(5)	3.2380(3)	I(5)−Ag(7)	2.9967(18)
Ag(4)−I(8)#1	2.7680(2)	I(6)−Ag(7)	2.7886(17)
Ag(4)−Ag(5)	2.9610(2)	I(7)−Ag(5)#2	2.7201(17)
Ag(5)−I(7)#1	2.7201(17)	I(8)−Ag(4)#2	2.7680(2)
Ag(5)−I(8)#1	2.8374(18)	I(8)−Ag(5)#2	2.8375(18)
Ag(6)−I(9)	2.7519(18)	Ni(1)−N(1)	2.1010(11)
Ag(6)−Ag(7)	2.9910(2)	Ni(1)−N(2)	2.0860(10)
Ag(7)−I(8)	2.7892(17)	Ni(1)−N(3)	2.0310(9)
Ag(7)−I(9)	2.7966(17)	Ni(1)−N(4)	2.0800(10)
I(1)−Ag(1)	2.6972(19)	Ni(1)−N(5)	2.0530(10)
I(1)−Ag(2)	2.7840(17)	Ni(1)−N(6)	2.0760(10)
I(1)−Ag(1)−I(2)	99.24(6)	I(8)#1−Ag(5)−I(3)	113.33(6)
I(1)−Ag(1)−I(4)#1	130.84(7)	I(3)−Ag(6)−I(5)	98.59(5)
I(1)−Ag(1)−I(5)#1	123.72(7)	I(4)−Ag(6)−I(3)	97.34(5)
I(4)#1−Ag(1)−I(2)	97.13(6)	I(4)−Ag(6)−I(5)	100.79(6)
I(4)#1−Ag(1)−I(5)#1	102.86(6)	I(9)−Ag(6)−I(3)	116.06(7)
I(5)#1−Ag(1)−I(2)	87.86(5)	I(9)−Ag(6)−I(4)	124.57(6)
I(1)−Ag(2)−I(2)	100.10(5)	I(9)−Ag(6)−I(5)	115.07(6)
I(1)−Ag(2)−I(3)	116.59(6)	I(6)−Ag(7)−I(8)	113.48(5)
I(1)−Ag(2)−I(6)	111.97(6)	I(6)−Ag(7)−I(9)	115.08(6)
I(3)−Ag(2)−I(2)	109.90(6)	I(8)−Ag(7)−I(9)	111.72(5)
I(6)−Ag(2)−I(2)	108.11(5)	I(6)−Ag(7)−I(5)	103.59(5)
I(6)−Ag(2)−I(3)	109.54(5)	I(8)−Ag(7)−I(5)	99.63(5)
I(2)−Ag(3)−I(5)	95.39(5)	I(9)−Ag(7)−I(5)	111.95(5)
I(6)−Ag(3)−I(2)	112.42(5)	N(2)−Ni(1)−N(1)	78.6(4)
I(6)−Ag(3)−I(5)	103.66(6)	N(3)−Ni(1)−N(1)	101.8(4)
I(7)−Ag(3)−I(2)	110.48(6)	N(3)−Ni(1)−N(2)	89.3(4)
I(7)−Ag(3)−I(5)	105.50(6)	N(3)−Ni(1)−N(4)	78.6(4)
I(7)−Ag(3)−I(6)	124.57(7)	N(3)−Ni(1)−N(5)	92.8(4)
I(2)−Ag(4)−I(3)	116.92(6)	N(3)−Ni(1)−N(6)	166.6(4)
I(2)−Ag(4)−I(8)#1	121.69(6)	N(4)−Ni(1)−N(1)	177.2(4)
I(8)#1−Ag(4)−I(3)	121.18(6)	N(4)−Ni(1)−N(2)	98.6(4)
I(2)−Ag(4)−I(5)	92.81(6)	N(4)−Ni(1)−N(6)	90.6(4)
I(3)−Ag(4)−I(5)	93.25(6)	N(5)−Ni(1)−N(1)	92.3(4)
I(8)#1−Ag(4)−I(5)	88.61(6)	N(5)−Ni(1)−N(2)	170.9(4)
I(7)#1−Ag(5)−I(4)	117.24(6)	N(5)−Ni(1)−N(4)	90.4(4)
I(7)#1−Ag(5)−I(8)#1	120.12(6)	N(5)−Ni(1)−N(6)	79.2(4)
I(8)#1−Ag(5)−I(4)	99.86(5)	N(6)−Ni(1)−N(1)	89.3(4)
I(4)−Ag(5)−I(3)	93.35(5)	N(6)−Ni(1)−N(2)	100.4(4)
I(7)#1−Ag(5)−I(3)	109.64(6)		

Symmetry transformations used to generate equivalent atoms: #1 $-x+3/2$, $y+1/2$, $-z+1/2$; #2 $-x+3/2$, $y-1/2$, $-z+1/2$.

Table S6 Hydrogen bonds (Å) and angles (º) for compound **2**.

C−H···I	*d*(C−H)	*d*(H···I)	*d*(C···I)	<(CHI)
C(8)−H(8)···I(9)#3	0.93	3.04	3.857(13)	147.1
C(17)−H(17)···I(1)#4	0.93	3.32	3.912(13)	123.2
C(29)−H(29) ··I(4)#2	0.93	3.15	3.902(13)	139.0

Symmetry transformations used to generate equivalent atoms: #3 *x*+1, *y*, *z*; #4 −*x*+2, −*y*+1, −*z*+1.

Table S7 C−H···π interactions (Å and ˚) for compound **2**.

C−H(I)···Cg	*d*(H···Cg)	*d*(C···Cg)	∠(C−H···Cg)
C(25)−H(25)···Cg(1)#5	2.97	3.737(15)	141
C(1)−H(1)···Cg(2)#5	2.95	3.738(15)	143
C(23)−H(23)···Cg(3)#6	2.87	3.591(15)	136

Symmetry codes: #5 *x*, *y*, *z*; #6 1/2−*x*, 1/2+*y*, 1/2−*z*. Cg(1): N(3)−C(13)−C(14)−C(16)−C(17)−C(18); Cg(2): N(5)−C(25)−C(27)−C(28)−C(29)−C(30); Cg(3): N(6)−C(31)−C(32)−C(33)−C(34)−C(35).

Table S8 Selected bond lengths (Å) and bond angles (°) for compound **3**.

I(1)−Ag(1)	2.6924(19)	I(9)−Ag(6)#4	2.701(9)
I(1)−Ag(2)	2.877(3)	I(9)−Ag(5)	3.058(3)
I(2)−Ag(2)#1	2.935(3)	Ag(1)−I(1)#1	2.6924(19)
I(2)−Ag(3)	2.997(3)	Ag(1)−Ag(2)	2.937(3)
I(2)−Ag(1)	3.084(3)	Ag(1)−Ag(2)#1	2.937(3)
I(2)−Ag(2)	3.125(3)	Ag(1)−I(2)#1	3.084(3)
I(3)−Ag(2)	2.734(3)	Ag(2)−I(2)#1	2.935(3)
I(3)−Ag(3)	2.833(3)	Ag(2)−Ag(3)	3.000(3)
I(4)−Ag(3)#1	2.746(9)	Ag(3)−I(4A)#1	2.88(3)
I(4)−Ag(3)	2.746(9)	Ag(4)−I(6)#3	2.822(4)
I(4A)−Ag(3)	2.63(3)	Ag(5)−Ag(6)	2.772(8)
I(4A)−Ag(3)#1	2.88(3)	Ag(5)−Ag(6)#4	2.852(8)
I(5)−Ag(3)	2.817(2)	Ag(5)−I(7)#4	2.900(3)
I(5)−Ag(3)#2	2.817(2)	Ag(6)−Ag(6)#4	0.844(10)
I(6)−Ag(4)#3	2.822(4)	Ag(6)−I(9)#4	2.701(9)
I(6)−Ag(4)	2.829(3)	Ag(6)−Ag(5)#4	2.852(8)
I(7)−Ag(6)	2.873(5)	Co(1)−N(4)	1.907(16)
I(7)−Ag(5)#4	2.900(3)	Co(1)−N(5)	1.911(15)
I(7)−Ag(4)	2.995(3)	Co(1)−N(1)	1.919(14)
I(7)−Ag(5)	3.081(3)	Co(1)−N(3)	1.923(15)
I(8)−Ag(5)	2.687(3)	Co(1)−N(2)	1.929(17)
I(8)−Ag(4)	2.887(3)	Co(1)−N(6)	1.941(15)
I(9)−Ag(6)	2.694(9)		
I(1)−Ag(1)−I(1)#1	143.81(15)	I(6)#3−Ag(4)−I(7)	109.99(10)
I(1)−Ag(1)−I(2)#1	101.10(6)	I(6)−Ag(4)−I(7)	102.77(10)
I(1)#1−Ag(1)−I(2)#1	104.82(6)	I(8)−Ag(4)−I(7)	101.36(10)
I(1)−Ag(1)−I(2)	104.82(6)	I(8)−Ag(5)−I(7)#4	134.09(12)
I(1)#1−Ag(1)−I(2)	101.10(6)	I(8)−Ag(5)−I(9)	115.55(11)
I(2)#1−Ag(1)−I(2)	87.61(10)	I(7)#4−Ag(5)−I(9)	93.01(9)
I(3)−Ag(2)−I(1)	122.96(10)	I(8)−Ag(5)−I(7)	103.96(11)
I(3)−Ag(2)−I(2)#1	127.09(10)	I(7)#4−Ag(5)−I(7)	102.93(9)
I(1)−Ag(2)−I(2)#1	100.46(8)	I(9)−Ag(5)−I(7)	103.64(9)
I(3)−Ag(2)−I(2)	109.21(8)	I(9)−Ag(6)−I(9)#4	136.23(18)
I(1)−Ag(2)−I(2)	99.51(8)	I(9)−Ag(6)−I(7)	120.2(3)
I(2)#1−Ag(2)−I(2)	89.54(7)	I(9)#4−Ag(6)−I(7)	101.7(3)
I(4A)−Ag(3)−I(5)	110.5(7)	N(4)−Co(1)−N(5)	92.8(7)
I(4)−Ag(3)−I(5)	104.45(17)	N(4)−Co(1)−N(1)	94.0(6)
I(4A)−Ag(3)−I(3)	113.7(10)	N(5)−Co(1)−N(1)	95.2(6)
I(4)−Ag(3)−I(3)	124.62(9)	N(4)−Co(1)−N(3)	83.9(7)

I(5)−Ag(3)−I(3)	107.40(8)	N(5)−Co(1)−N(3)	176.3(6)
I(4A)−Ag(3)−I(4A)#1	21.2(19)	N(1)−Co(1)−N(3)	86.6(6)
I(5)−Ag(3)−I(4A)#1	99.8(7)	N(4)−Co(1)−N(2)	178.2(6)
I(3)−Ag(3)−I(4A)#1	134.9(9)	N(5)−Co(1)−N(2)	88.2(6)
I(4A)−Ag(3)−I(2)	119.6(8)	N(1)−Co(1)−N(2)	84.5(6)
I(4)−Ag(3)−I(2)	112.04(19)	N(3)−Co(1)−N(2)	95.1(6)
I(5)−Ag(3)−I(2)	93.07(7)	N(4)−Co(1)−N(6)	87.8(6)
I(3)−Ag(3)−I(2)	110.15(9)	N(5)−Co(1)−N(6)	83.1(6)
I(4A)#1−Ag(3)−I(2)	103.4(7)	N(1)−Co(1)−N(6)	177.6(6)
I(6)#3−Ag(4)−I(6)	102.86(10)	N(3)−Co(1)−N(6)	95.2(6)
I(6)#3−Ag(4)−I(8)	116.16(11)	N(2)−Co(1)−N(6)	93.7(6)
I(6)−Ag(4)−I(8)	122.62(11)		

Symmetry transformations used to generate equivalent atoms: #1 −*x*+1, *y*, −*z*+1/2; #2 −*x*+1, −*y*+2, −*z*; #3 −*x*+2, −*y*+1, −*z*; #4 −*x*+2, *y*, −*z*+1/2.

Table S9 Hydrogen bonds (Å) and angles (º) for compound **3**.

C−H···I	*d*(C−H)	*d*(H···I)	*d*(C···I)	<(CHI)
C(4)−H(4)···I(6)	0.93	3.03	3.71(2)	131.0
C(5)−H(5)···I(8)	0.93	3.16	3.80(3)	127.6
C(8)−H(8)···I(8)	0.93	3.23	3.94(3)	134.8
C(17)−H(17)···I(5)#5	0.93	3.09	4.00(2)	169.1
C(20)−H(20)···I(5)#5	0.93	2.95	3.87(3)	172.5
C(29)−H(29)···I(9)#6	0.93	3.04	3.767(19)	135.9

Symmetry transformations used to generate equivalent atoms: #5 *x*, *y*−1, *z*; #6 *x*, −*y*+1, *z*+1/2.

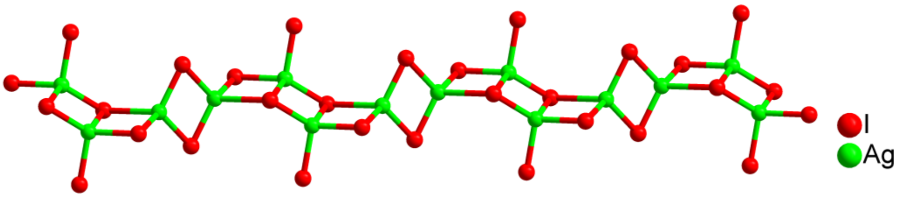

Figure S1 The $[Ag_2I_4]_n^{2n-}$ chain in compound **1**.

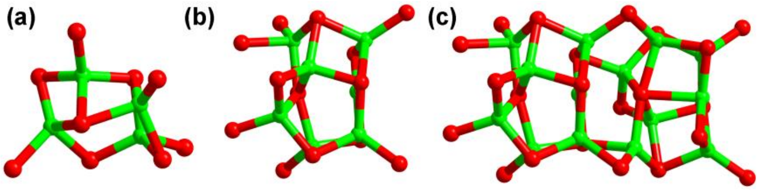

Figure S2 The $[Ag_4I_9]$ unit (a), $[Ag_7I_{13}]$ unit (b) and $[Ag_{14}I_{22}]$ unit (c) in compound **2**.

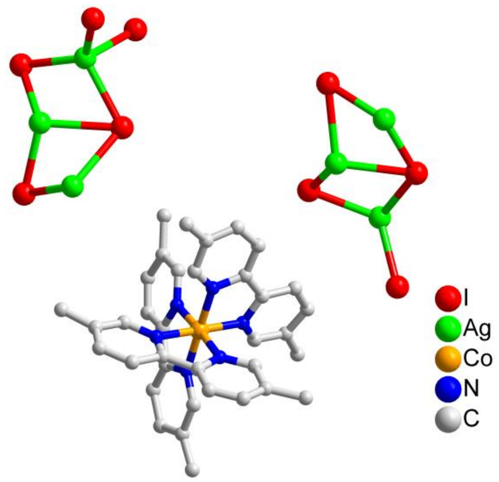

Figure S3 The asymmetric unit of compound **3**.

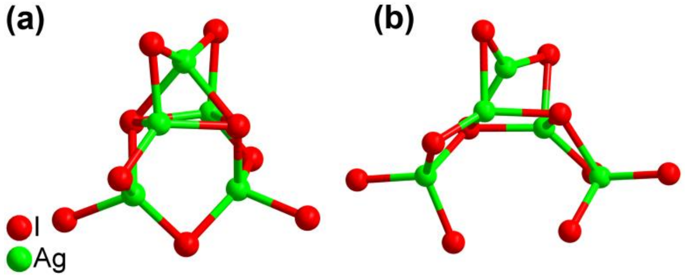

Figure S4 The $[Ag_5I_9]$ unit (a) and $[Ag_5I_{10}]$ unit (b) in compound **3**.

2. Hirshfeld surface analyses

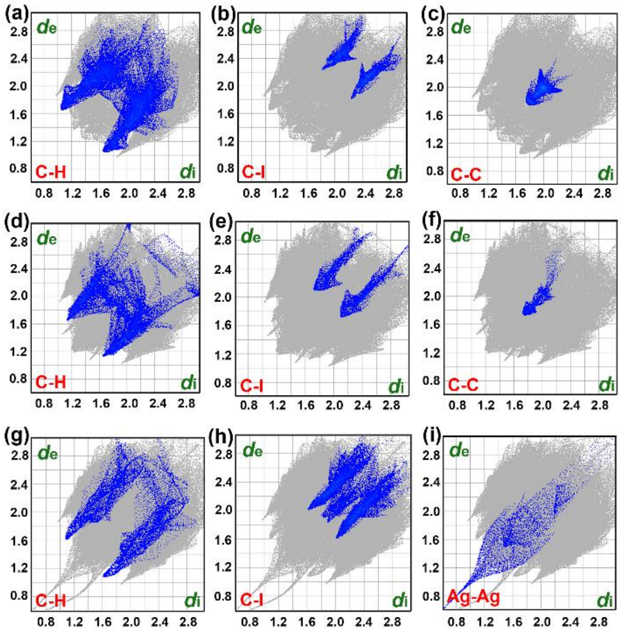

Figure S5 Fingerprint plots: resolved into C···H (a), C···I (b) and C···C (c) for compound **1**; resolved into C···H (d), C···I (e) and C···C (f) for compound **2**; resolved into C···H (g), C···I (h) and Ag···Ag (i) for compound **3**.

Table S10 The Hirshfeld surface comparisons of compounds **1**−**3** with some related analogues.

Compounds	Weak interactions (< 4.0 Å; X = I, Br)				
	C−H···X (%)	C−H···π (%)	π···π (%)	X···π (%)	H···H (%)
Com-1	√ (35.1)	√ (17.2)	√ (3.2)	√ (5.0)	√ (20.7)
Com-2	√ (32.0)	√ (9.2)	√ (1.1)	√ (2.8)	√ (31.0)
Com-3	√ (40.8)	√ (5.2)	×	√ (9.5)	√ (26.2)
$[Ni(phen)_3]Ag_2PbI_6$ [1]	√ (27.6)	√ (10.0)	√ (16.0)	√ (20.0)	√ (13.9)
$[Fe(phen)_3]_2Ag_3Pb_2I_{11}$ [2]	√ (36.1)	√ (14.0)	√ (1.9)	√ (10.2)	√ (19.5)
$[Fe(bipy)_3]AgBiI_6$ [3]	√ (46.4)	√ (15.3)	√ (1.4)	√ (5.0)	√ (24.1)
$[Co(bipy)_3]_2Ag_4Bi_2I_{16}$ [4]	√ (55.9)	√ (9.3)	√ (0.8)	√ (9.7)	√ (17.8)
$[NH_4][Fe(bipy)_3]_2Ag_6Br_{11}$ [5]	√ (30.9)	√ (15.5)	√ (2.2)	×	√ (23.8)
$[Ni(5,5\text{-dmbpy})_3]_2Ag_{4.9}I_{8.9}\cdot 4H_2O$ [6]	√ (27.8)	√ (13.8)	√ (0.7)	√ (3.1)	√ (32.0)

3. Physical measurements

3a). PXRD

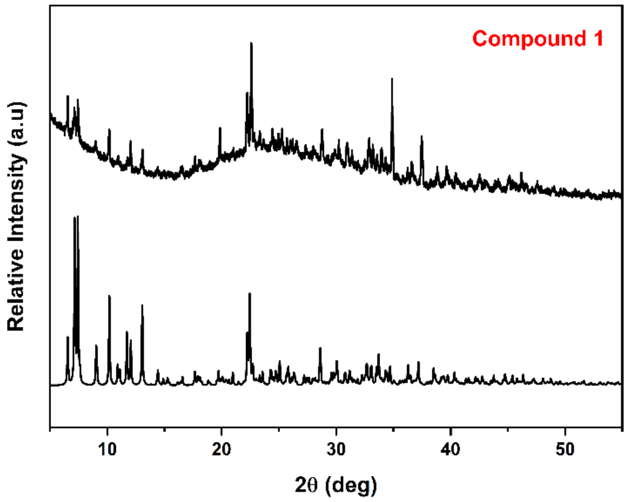

Figure S6 Experimental and simulated PXRD patterns of compound **1**.

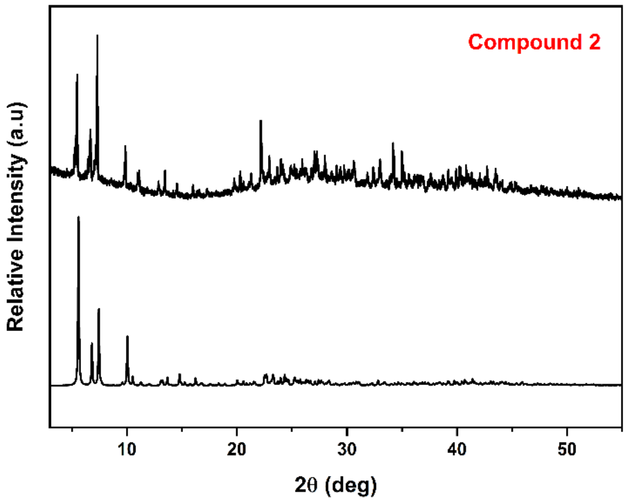

Figure S7 Experimental and simulated PXRD patterns of compound **2**.

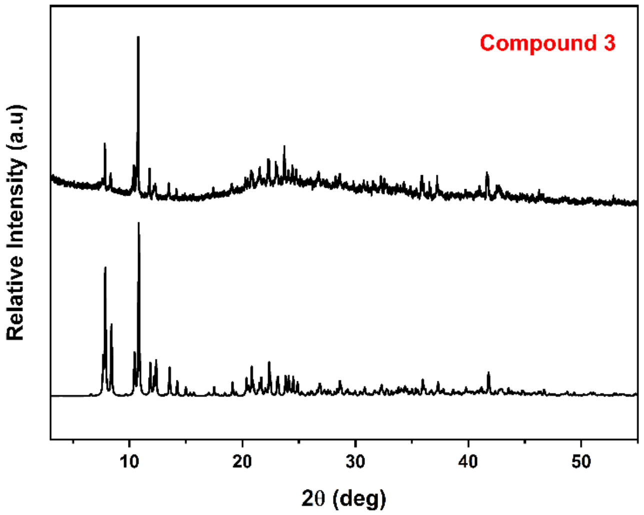

Figure S8 Experimental and simulated PXRD patterns of compound **3**.

3b) EDX

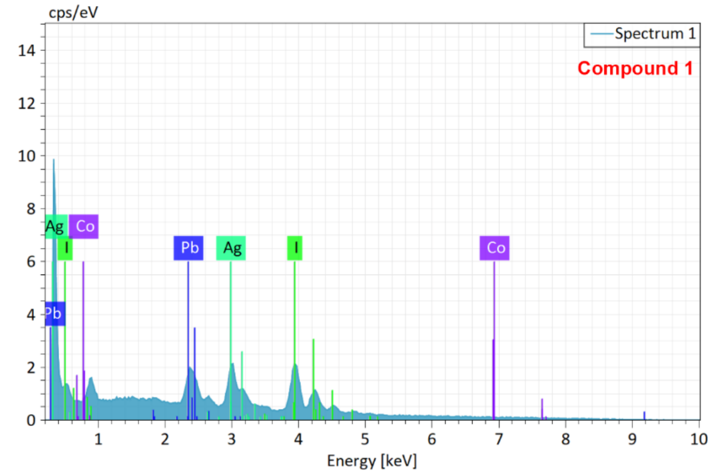

Figure S9 EDX spectrum of compound **1**.

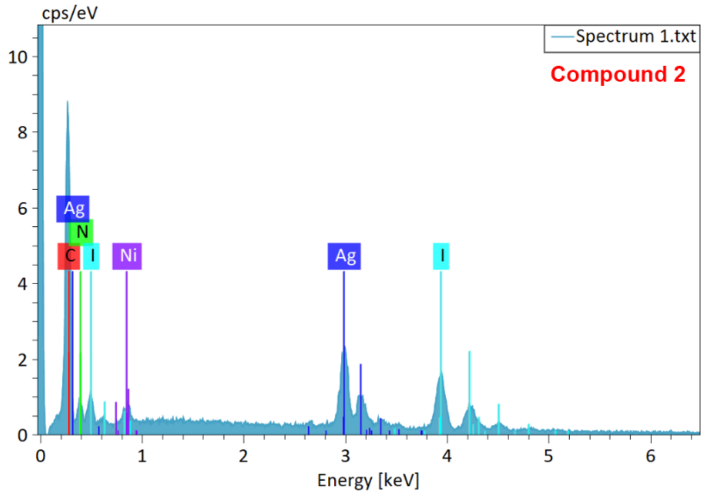

Figure S10 EDX spectrum of compound **2**.

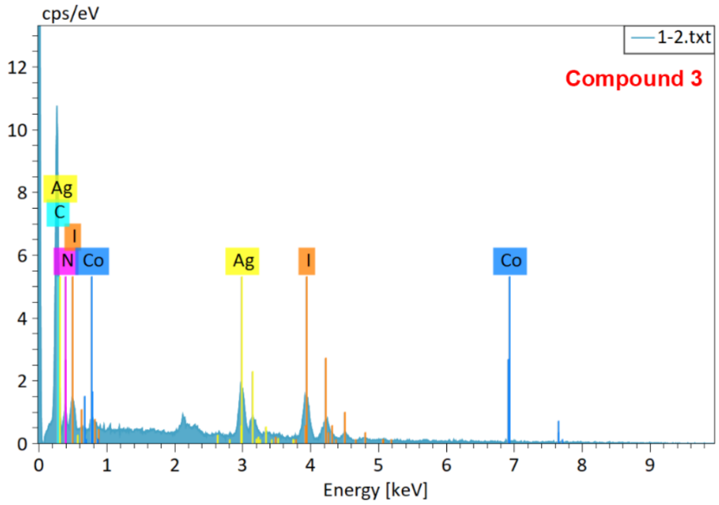

Figure S11 EDX spectrum of compound **3**.

3c) TGA

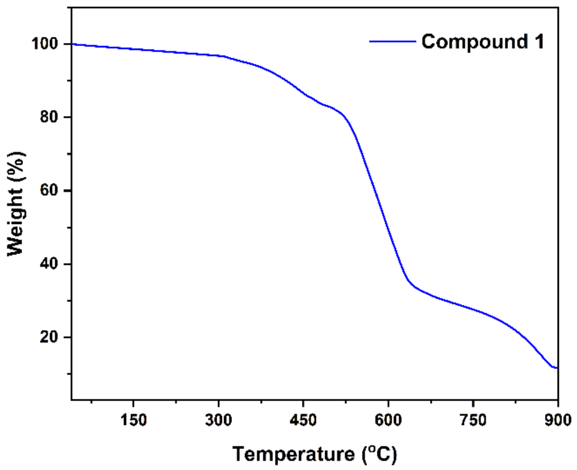

Figure S12 TGA curve of compound **1**.

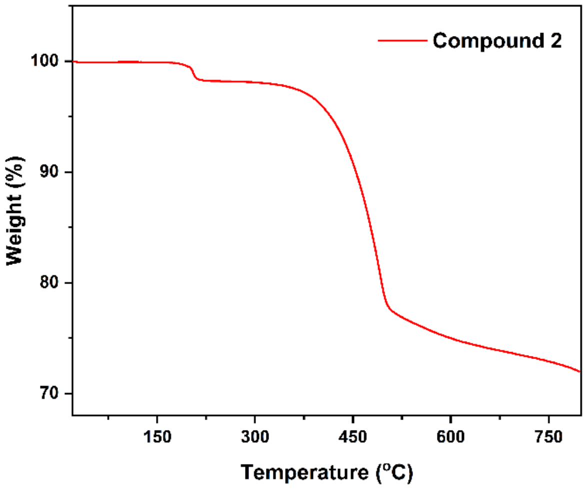

Figure S13 TGA curve of compound **2**.

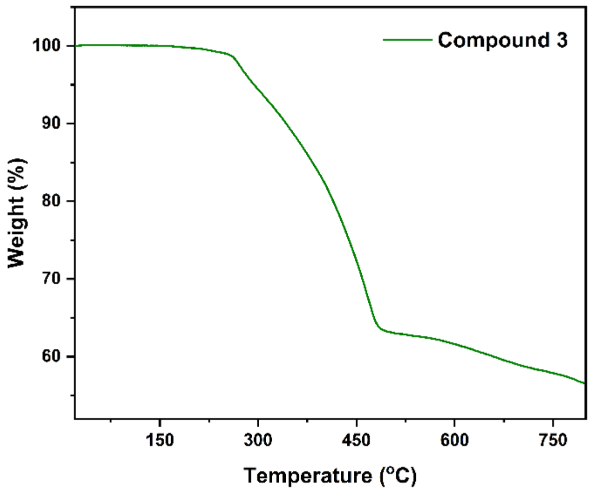

Figure S14 TGA curve of compound **3**.

4. Theoretical calculations

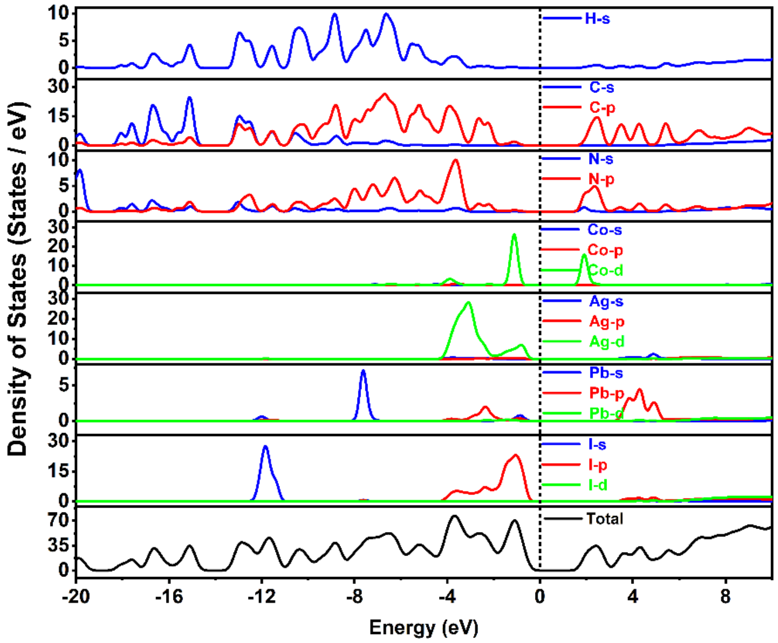

Figure S15 Total density of states and partial density of states for compound **1**. The Fermi level is set at 0 eV (dotted line).

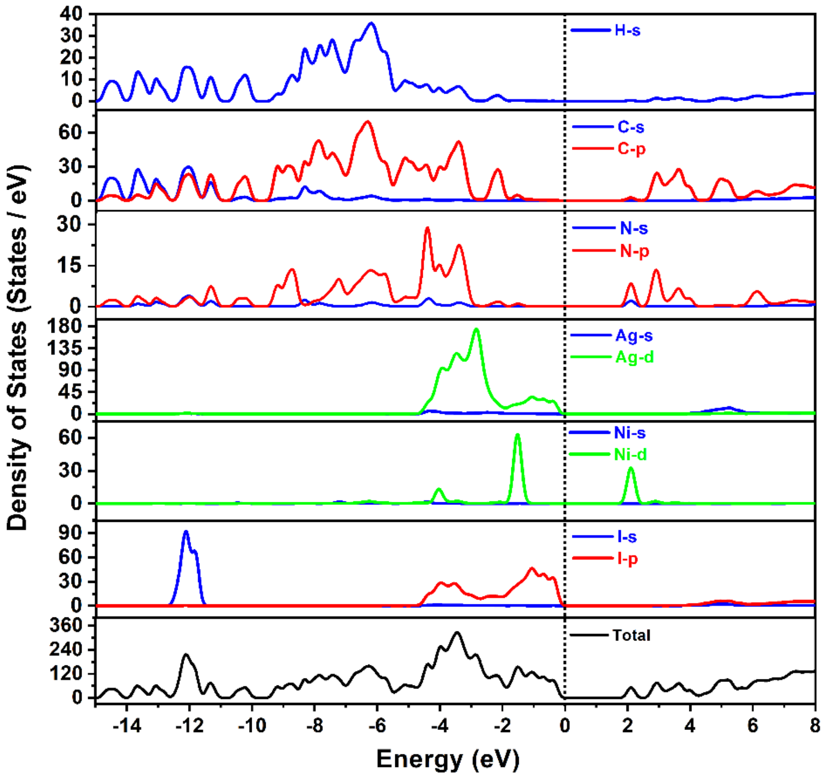

Figure S16 Total density of states and partial density of states for compound **2**. The Fermi level is set at 0 eV (dotted line).

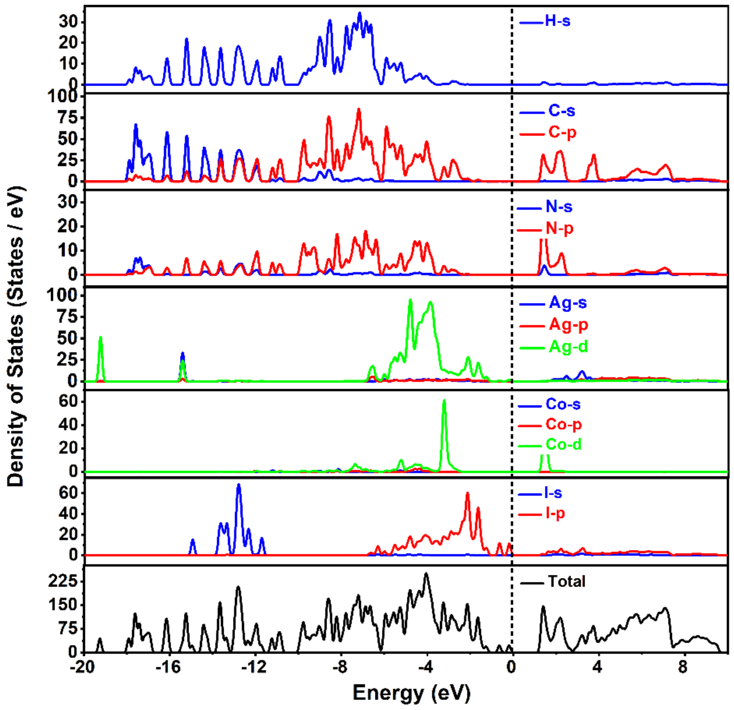

Figure S17 Total density of states and partial density of states for compound **3**. The Fermi level is set at 0 eV (dotted line).

Table S11 The structural comparisons of compounds **1−3** with some related analogues.

Compounds	Space group	Dimension	Basic building block	Halogen
Com-1	*P*-1	1D	$[PbI_4]$; $[AgI_4]$	μ-1; μ-2; μ-3
Com-2	$P2_1/n$	1D	$[AgI_3]$; $[AgI_4]$	μ-2; μ-3; μ-4
Com-3	$P2/c$	1D	$[AgI_3]$; $[AgI_4]$	μ-2; μ-4
$[Ni(phen)_3]Ag_2PbI_6$ [1]	*P*-1	1D	$[PbI_4]$; $[AgI_4]$	μ-1; μ-2; μ-3
$[Fe(phen)_3]_2Ag_3Pb_2I_{11}$ [2]	*P*-1	1D	$[PbI_5]$; $[AgI_2]$; $[AgI_4]$	μ-1; μ-2; μ-3
$[Zn(bipy)_3]AgPb_2I_7$ [7]	$P2_1/c$	1D	$[PbI_4]$; $[PbI_6]$; $[AgI_4]$	μ-1; μ-2; μ-3
$[Ni(en)_2]Ag_2PbI_6$ [8]	$Pca2_1$	2D	$[PbI_6]$; $[AgI_4]$	μ-2; μ-3
$[Co(bipy)_3]Ag_3I_6$ [9]	*R*-3	0D	$[AgI_4]$	μ-1; μ-3
$[Co(phen)_3]Ag_2I_4\cdot 3DMF$ [10]	*P*-1	0D	$[AgI_4]$	μ-1; μ-2; μ-3
$[Co(bipy)_3]Ag_5I_7$ [11]	$P2_12_12_1$	1D	$[AgI_4]$	μ-2; μ-3; μ-5
$[Mn(phen)_3]Ag_5I_7$ [12]	*P*-1	2D	$[AgI_4]$	μ-2; μ-3; μ-4
$[Cd(phen)_3]_2Ag_{13}I_{17}$ [13]	$P2_13$	3D	$[AgI_4]$	μ-2; μ-4
$K[Fe(bipy)_3]_2Ag_6I_{11}$ [14]	*R*-3	2D	$[AgI_4]$	μ-1; μ-2; μ-3
$[Mn(bipy)_3]Ag_3I_5$ [15]	$P2_12_12_1$	1D	$[AgI_4]$	μ-1; μ-2; μ-3

Reference:

[1] Ren, X.C.; Li, J.; Wang, W.H.; Shao, Y.N.; Zhang, B.; Li, L.Z. Hybrid silver haloplumbates containing metal complexes: Syntheses, structures and photoelectric properties. *J. Solid State Chem.* **2022**, *308*, 122912.

[2] Pang, M.; Ren, X.C.; Chen, X.; Shen, H.Y.; Li, J.; Zhang, B.; Li, L.Z. Two heterometallic silver-iodoplumbates with $[Fe(phen)_3]^{2+}$ complexes: Syntheses, structures, photocurrent responses and theoretical studies. *Inorg. Chem. Commun.* **2022**, *139*, 109342.

[3] Zhang, B.; Li, J.; Pang, M.; Wang, Y.S.; Liu, M.Z.; Zhao, H.M. Four discrete silver iodobismuthates/bromobismuthates with metal complexes: Syntheses, structures, photocurrent responses, and theoretical studies. *Inorg. Chem.* **2022**, *61*, 406−413.

[4] Zhang, B.; Li, J.; Pang, M.; Chen, X.; Liu, M.Z. Two $[Co(bipy)_3]^{3+}$-templated silver halobismuthate hybrids: Syntheses, structures, photocurrent responses, and theoretical studies. *Inorg. Chem.* **2022**, *61*, 9808−9815.

[5] Zhang, B.; Li, J.; Chen, X.; Yang, M.F.; Shen, H.Y.; Zhu, J.C. $[NH_4][Fe(bipy)_3]_2[Ag_6Br_{11}]$: Synthesis, structure, characterization and photocurrent response. *Inorg. Chem. Commun.* **2022**, *137*, 109250.

[6] Zhang, B.; Li, W.A.; Li, J.; Xu, Y.P.; Xu, Y.R.; Wang, W.H.; Zou, G.D. [Ni(5,5'-

dmbpy)$_3$]$_2$Ag$_{4.9}$I$_{8.9}$·4H$_2$O: A discrete iodoargentate with transition metal complexes. *Inorg. Chem. Commun.* **2020**, *121*, 108219.

[7] Yue, C.Y.; Lei, X.W.; Lu, X.X.; Li, Y.; Wei, J.C.; Wang, W.; Yin, Y.D.; Wang, N. Comparison studies of hybrid lead halide $[MPb_2X_7]^{2-}$ (M = Cu, Ag; X = Br, I) chains: Band structures and visible light driven photocatalytic properties. *Dalton Trans.* **2017**, *46*, 9235−9244.

[8] Zheng, W.; Chen, N.N.; Gao, Y.; Wu, B.; Jia, D.X. Heterometallic Pb−Ag iodides from 1-D chains to 2-D layers induced by transition metal complex cations: Syntheses, crystal structures, and photocatalytic properties. *Eur. J. Inorg. Chem.* **2019**, *2019*, 4752−4759.

[9] Tang, C.; Sun, Y.; Liu, J.; Xu, Q.; Zhang, C.Y. [Co(2,2'-bipy)$_3$]Ag$_3$I$_6$ with a hole structure facilitates dye adsorption and photocatalytic reduction. *Dalton Trans.* **2022**, *51*, 16784−16789.

[10] Tang, C.; Yao, J.; Li, Y.; Xia, Z.; Liu, J.; Zhang, C.Y. Transition-metal-complex-directed synthesis of hybrid iodoargentates with single-crystal to single-crystal structural transformation and photocatalytic properties. *Inorg. Chem.* **2020**, *59*, 13962−13971.

[11] Lei, X.W.; Yue, C.Y.; Wu, F.; Jiang, X.Y.; Chen, L.N. Syntheses, crystal structures and photocatalytic properties of transition metal complex directed iodoargentates: [TM(2,2-bipy)$_3$]Ag$_5$I$_7$. *Inorg. Chem. Commun.* **2017**, *77*, 64−67.

[12] Lei, X.W.; Yue, C.Y.; Feng, L.J.; Han, Y.F.; Meng, R.R.; Yang, J.T.; Ding, H.; Gao, C.S.; Wang, C.Y. Syntheses, crystal structures and photocatalytic properties of four hybrid iodoargentates with zero- and two-dimensional structures. *CrystEngComm* **2016**, *18*, 427−436.

[13] Yu, T.; Fu, Y.; Wang, Y.; Hao, P.; Shen, J.; Fu, Y. Hierarchical symmetry transfer and flexible charge matching in five $[M(phen)_3]^{2+}$ directed iodoargentates with 1 to 3D frameworks. *CrystEngComm* **2015**, *17*, 8752−8761.

[14] Lei, X.W.; Yue, C.Y.; Zhao, J.Q.; Han, Y.F.; Yang, J.T.; Meng, R.R.; Gao, C.S.; Ding, H.; Wang, C.Y.; Chen, W.D.; Hong, M.C. Two types of 2D layered iodoargentates based on trimeric Ag$_3$I$_7$ secondary building units and hexameric Ag$_6$I$_{12}$ ternary building units: Syntheses, crystal structures, and efficient visible light responding photocatalytic properties. *Inorg. Chem.* **2015**, *54*, 10593−10603.

[15] Lei, X.W.; Yue, C.Y.; Zhao, J.Q.; Han, Y.F.; Ba, Z.R.; Wang, C.; Liu, X.Y.; Gong, Y.P.; Liu, X.Y. Syntheses, crystal structures, and photocatalytic properties of polymeric iodoargentates [TM(2,2-bipy)]$_3$Ag$_3$I$_5$ (TM = Mn, Fe, Co, Ni, Zn). *Eur. J. Inorg. Chem.* **2015**, 4412−4419.
